# Loss of atrial natriuretic peptide signaling causes insulin resistance, mitochondrial dysfunction, and low endurance capacity

**DOI:** 10.1126/sciadv.adl4374

**Published:** 2024-10-09

**Authors:** Deborah Carper, Marlène Lac, Marine Coue, Axel Labour, Andre Märtens, Jorge Alberto Ayala Banda, Laurène Mazeyrie, Mie Mechta, Lars Roed Ingerslev, Mohamed Elhadad, Justine Vily Petit, Claire Maslo, Laurent Monbrun, Peggy Del Carmine, Yannis Sainte-Marie, Virginie Bourlier, Claire Laurens, Gilles Mithieux, Denis R. Joanisse, Charles Coudray, Christine Feillet-Coudray, Emilie Montastier, Nathalie Viguerie, Geneviève Tavernier, Melanie Waldenberger, Annette Peters, Rui Wang-Sattler, Jerzy Adamski, Karsten Suhre, Christian Gieger, Gabi Kastenmüller, Thomas Illig, Ralf Lichtinghagen, Jochen Seissler, Remy Mounier, Karsten Hiller, Jens Jordan, Romain Barrès, Michaela Kuhn, Dominik Pesta, Cedric Moro

**Affiliations:** ^1^ Institute of Metabolic and Cardiovascular Diseases, INSERM/Paul Sabatier University, UMR1297, Team MetaDiab, Toulouse, France.; ^2^ Department of Bioinformatics and Biochemistry, Braunschweig Integrated Centre of Systems Biology (BRICS), Technische Universität Braunschweig and Physikalisch-Technische Bundesanstalt, Brunswick, Germany.; ^3^ Novo Nordisk Foundation Center for Basic Metabolic Research, Faculty of Health and Medical Sciences, University of Copenhagen, Copenhagen, Denmark.; ^4^ Institute of Epidemiology, Helmholtz Zentrum München, German Research Center for Environmental Health (GmbH), Neuherberg, Germany.; ^5^ INSERM U1213, Nutrition, Diabète et Cerveau, Lyon, France.; ^6^ Institut NeuroMyoGène, Université Claude Bernard Lyon 1, INSERM U1315, CNRS UMR, 5261 Lyon, France.; ^7^ Department of Kinesiology, Centre de Recherche de l’Institut Universitaire de Cardiologie et de Pneumologie de Québec, Université Laval, Québec, Canada.; ^8^ Dynamique Musculaire Et Métabolisme, INRAE, UMR866, Université Montpellier, Montpellier, France.; ^9^ Institute of Experimental Genetics, Helmholtz Zentrum München, German Research Center for Environmental Health, Ingolstädter Landstraße 1, 85764 Neuherberg, Germany.; ^10^ Department of Biochemistry, Yong Loo Lin School of Medicine, National University of Singapore, 8 Medical Drive, Singapore 117597, Singapore.; ^11^ Institute of Biochemistry, Faculty of Medicine, University of Ljubljana, Vrazov trg 2, 1000 Ljubljana, Slovenia.; ^12^ Bioinformatics Core, Weill Cornell Medicine-Qatar, Doha, Qatar.; ^13^ Institute of Computational Biology, Helmholtz Zentrum München, German Research Center for Environmental Health, Neuherberg, Germany.; ^14^ Hannover Unified Biobank, Hannover Medical School, Hanover, Germany.; ^15^ Department of Clinical Chemistry, Hannover Medical School, Hannover, Germany.; ^16^ Diabetes Zentrum, Medizinische Klinik und Poliklinik IV, Klinikum der Universität München, LMU, München, Germany.; ^17^ Institute of Aerospace Medicine, German Aerospace Center, Cologne, Germany.; ^18^ Medical Faculty, University of Cologne, Cologne, Germany.; ^19^ Institute of Physiology, University of Würzburg, Würzburg, Germany.; ^20^ Center for Endocrinology, Diabetes, and Preventive Medicine (CEDP), University Hospital Cologne, Cologne, Germany.; ^21^ Cologne Excellence Cluster on Cellular Stress Responses in Aging-Associated Diseases (CECAD), University of Cologne, Cologne, Germany.

## Abstract

Type 2 diabetes (T2D) and obesity are strongly associated with low natriuretic peptide (NP) plasma levels and a down-regulation of NP guanylyl cyclase receptor-A (GCA) in skeletal muscle and adipose tissue. However, no study has so far provided evidence for a causal link between atrial NP (ANP)/GCA deficiency and T2D pathogenesis. Here, we show that both systemic and skeletal muscle ANP/GCA deficiencies in mice promote metabolic disturbances and prediabetes. Skeletal muscle insulin resistance is further associated with altered mitochondrial function and impaired endurance running capacity. ANP/GCA-deficient mice exhibit increased proton leak and reduced content of mitochondrial oxidative phosphorylation proteins. We further show that GCA is related to several metabolic traits in T2D and positively correlates with markers of oxidative capacity in human skeletal muscle. Together, these results indicate that ANP/GCA signaling controls muscle mitochondrial integrity and oxidative capacity in vivo and plays a causal role in the development of prediabetes.

## Teaser

Atrial natriuretic peptide signaling controls insulin sensitivity and skeletal muscle oxidative capacity in mice and humans.

## INTRODUCTION

Obesity is a major risk factor of type 2 diabetes (T2D) and is generally characterized by peripheral insulin resistance and a poor oxidative capacity in skeletal muscles (*1*). Obesity is also associated with neuroendocrine abnormalities. Epidemiological and Mendelian randomization studies demonstrated that obese patients and patients with T2D present low circulating levels of the cardiac hormone atrial natriuretic peptide (ANP) (*2*, *3*). ANP, a product of the *Nppa* gene, binds to a guanylyl cyclase receptor-A (GCA), encoded by the *Npr1* gene, to regulate blood volume and pressure (*4*). ANP is rapidly cleared from the bloodstream through its binding to a clearance receptor called natriuretic peptide receptor C (NPRC) encoded by the *Npr3* gene (*5*). Numerous prospective studies in large clinical cohorts revealed that individuals with low measured or genetically predicted ANP levels are at high risk of new onset T2D (*6*–*8*). Genetic variants of ANP or its biological receptor GCA are also related to T2D development (*9*). We and others further demonstrated reduced GCA expression in skeletal muscle and subcutaneous abdominal adipose tissue of obese patients and/or patients with T2D (*10*–*13*). Low adipose and skeletal muscle GCA expression was tightly correlated with insulin resistance (*11*, *14*). In contrast, high GCA gene expression in skeletal muscle associates with high oxidative capacity, and exercise training up-regulates GCA together with mitochondrial oxidative capacity in skeletal muscle of middle-aged obese individuals (*15*). Collectively, these observations suggest that the ANP/GCA system may play a key role in the pathogenesis of obesity-related metabolic disorders and T2D. Yet, it is still unclear whether ANP/GCA deficiency causes T2D and impairs muscle oxidative capacity, as well as what are the underlying molecular mechanisms.

We here took advantage of two complementary mouse models (*Nppa^−/−^* and *Npr1^+/−^*) to critically examine the causal link between ANP/GCA deficiency and the development of insulin resistance, poor oxidative capacity, and T2D. Both *Nppa^−/−^* and *Npr1^+/−^* mice displayed hallmarks of insulin resistance and prediabetes. We further demonstrate that muscle-specific knockdown of GCA signaling (MKD-*Gca*) is sufficient to cause prediabetes. This phenotype was associated with impaired insulin signaling, altered mitochondrial oxidative capacity, and decreased endurance running capacity in skeletal muscle. In humans, muscle GCA protein expression positively correlates with mitochondrial oxidative metabolism and oxidative capacity. Collectively, our data indicate that the ANP/GCA system plays a major role in the maintenance of insulin sensitivity and endurance capacity by controlling skeletal muscle oxidative capacity and mitochondrial integrity.

## RESULTS

### ANP deficiency promotes skeletal muscle insulin resistance in mice

We first examined the consequences of ANP deficiency on insulin and glucose tolerance in mice under standard (SD) and high-fat diet (HFD) nutritional conditions. Beforehand, *Nppa*^−/−^ mice were backcrossed on a C57BL/6J background, which are susceptible to diet-induced obesity and T2D. Cardiac *Nppa* mRNA levels were virtually null in *Nppa*^−/−^ mice compared to wild-type (WT) littermates *Nppa*^+/+^ (fig. S1A), while cardiac *Nppb* mRNA levels, the gene product of B-type natriuretic peptide (BNP), remained unaffected (fig. S1B). No change in *Npr1* and *Npr3* mRNA levels were found in skeletal muscle and adipose tissue of *Nppa*^−/−^ versus WT mice (fig. S1, C and D). As expected, a state of cardiac hypertrophy reflected by a 31% increase in heart weight to body weight ratio (*P* < 0.0001) (fig. S1E) and left ventricular (LV) mass (20%, *P* < 0.0001) (fig. S1F) was found in *Nppa*^−/−^ mice. However, this hypertrophy occurred in the absence of systolic dysfunction as LV ejection fraction (LVEF) remained unchanged in ANP-deficient mice (fig. S1G).

**Fig. 1.**
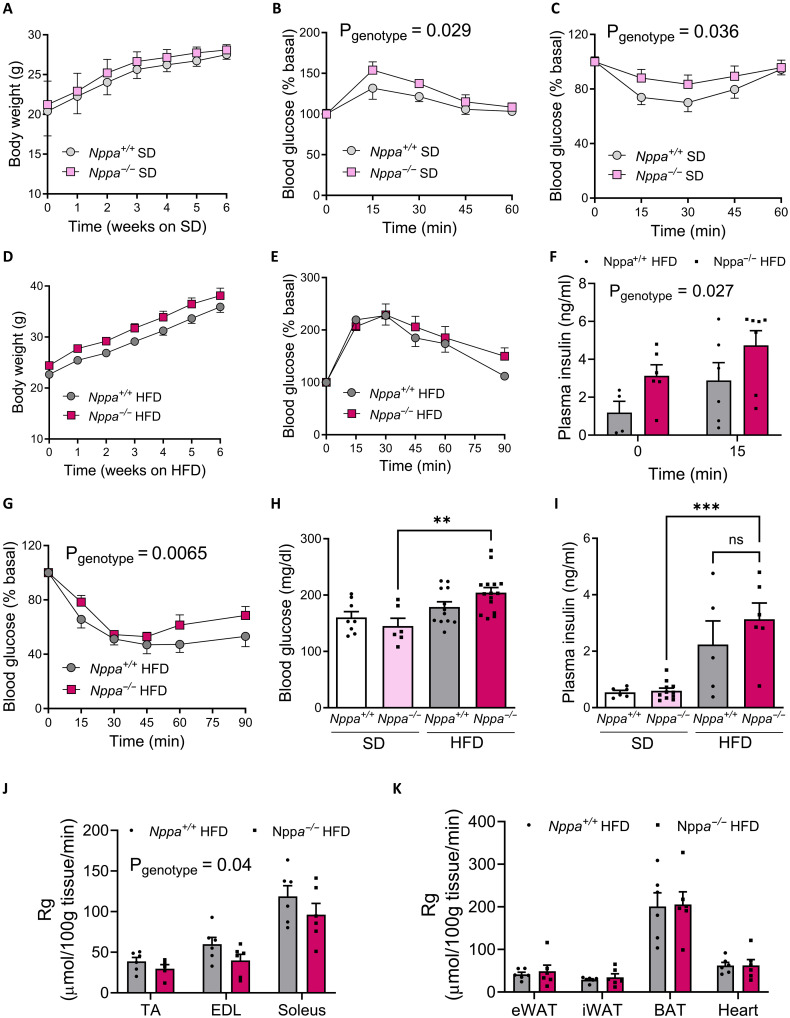
ANP deficiency promotes insulin resistance in lean and obese mice. Body weight (**A**) and blood glucose levels during a GTT (*n* = 8) (**B**) and an insulin tolerance test (ITT) (*n* = 13) (**C**) of *Nppa*^−/−^ and *Nppa*^+/+^ male mice fed SD. Body weight (**D**) and blood glucose levels during a GTT (**E**) and plasma insulin levels at t0 and t15 min of the GTT (**F**) in HFD-fed *Nppa*^−/−^ and *Nppa*^+/+^ mice. Blood glucose levels during an ITT (**G**) and fasting blood glucose (**H**) and fasting plasma insulin (**I**) levels under SD and HFD in *Nppa*^−/−^ and *Nppa*^+/+^ mice. Tissue-specific 2-DG uptake in the tibialis anterior, extensor digitorum longus (EDL), and soleus muscles (**J**) and in various adipose tissues and the heart (**K**) of *Nppa*^+/+^ and *Nppa*^−/−^ HFD-fed mice. **P < 0.01, ***P < 0.001, versus *Nppa*^+/+^.

We here show that ANP deficiency has no major influence on body weight gain (Fig. 1A) and body composition (fig. S1, H and I) under SD. However, glucose tolerance was slightly impaired (Fig. 1B), while insulin tolerance was blunted under SD (Fig. 1C). Similarly, ANP deficiency did not alter body weight gain (Fig. 1D) and glucose tolerance under HFD (Fig. 1E). However, *Nppa*^−/−^ mice fed HFD exhibited clear signs of whole-body insulin resistance reflected by elevated plasma insulin levels during the glucose tolerance test (GTT) (Fig. 1F) and reduced insulin sensitivity (Fig. 1G). These mice also had trends for elevated concentrations of fasting blood glucose (Fig. 1H), fasting insulin (Fig. 1I), and homeostasis model assessment for insulin resistance (HOMA-IR) (fig. S1J) compared to *Nppa*^+/+^ mice. We next investigated the tissue-specific glucose uptake in HFD-fed mice. Mice were injected with a mixed bolus of insulin and radiolabeled 2-deoxyglucose (2-DG) as previously described (*16*). We could further demonstrate a reduced glucose uptake (Rg) in all skeletal muscles investigated (Fig. 1J), while glucose uptake remained unchanged in other tissues (Fig. 1K). Together, these results indicate that ANP deficiency causes skeletal muscle insulin resistance in mice.

### GCA haploinsufficiency exacerbates HFD-induced insulin resistance in mice

Since previous data indicated that GCA protein expression is reduced by about half in adipose tissue and skeletal muscle of obese humans and mice (*10*, *14*), we next investigated the influence of *Npr1*^+/−^ haploinsufficiency on metabolic phenotypes and T2D pathogenesis. *Npr1*^+/−^ mice displayed a 45 and 47% decrease of *Npr1* mRNA expression in skeletal muscle and adipose tissue, respectively, compared to *Npr1*^+/+^ mice (fig. S2A), without any change in *Npr3* expression (fig. S2B). GCA protein expression was reduced by 48% in skeletal muscle (fig. S2C). As observed in *Nppa*^−/−^ mice, *Npr1*^+/−^ mice displayed a cardiac hypertrophy (fig. S2D) with a preserved LVEF (fig. S2E). Plasma ANP and BNP concentrations remained unchanged compared to *Npr1*^+/+^ mice (fig. S2, F and G).

**Fig. 2.**
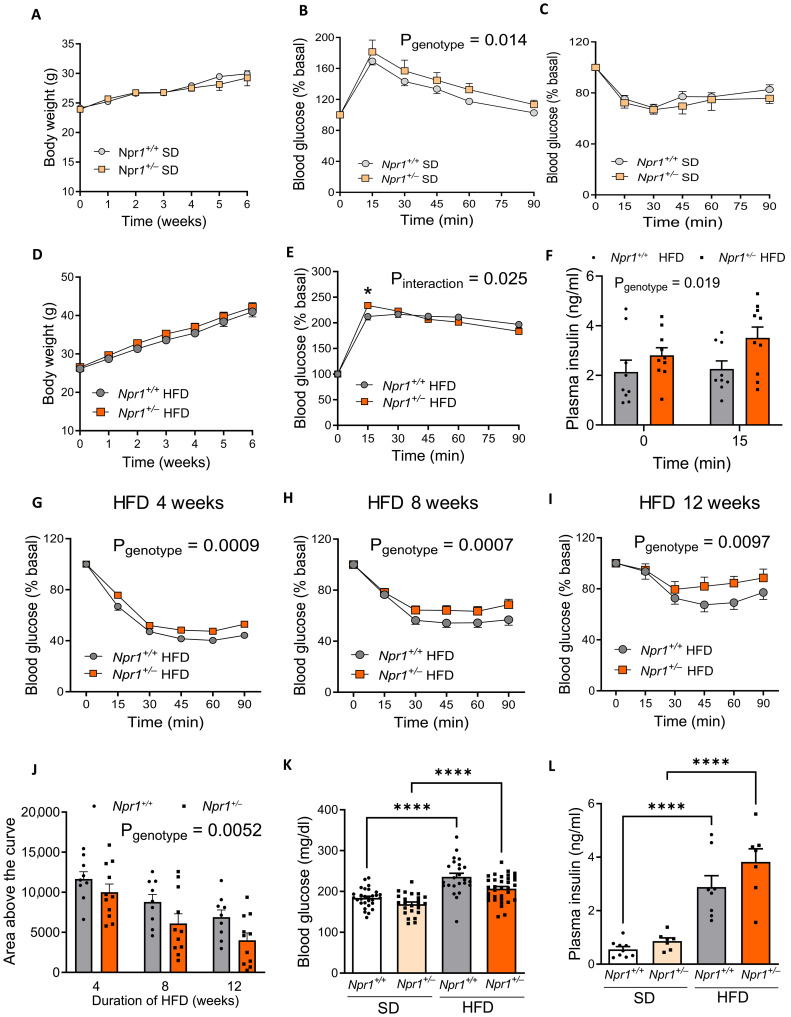
GCA haploinsufficiency exacerbates HFD-induced insulin resistance. Body weight (**A**) and blood glucose levels during a GTT (*n* = 7 to 9) (**B**) and an ITT (*n* = 17 to 21) (**C**) in *Npr1*^+/−^ and *Npr1*^+/+^ male mice fed an SD. Body weight (**D**) and blood glucose levels during a GTT (**E**) and plasma insulin levels at t0 and t15 min of the GTT (**F**) in *Npr1*^+/−^ and *Npr1*^+/+^ mice fed an HFD for 12 weeks (*n* = 8 to 14). Blood glucose levels during an ITT after 4 weeks (**G**), 8 weeks (**H**), and 12 weeks (**I**) of HFD in *Npr1*^+/−^ and *Npr1*^+/+^ mice (*n* = 17 to 25). Corresponding area above the curve of the ITT at 4, 8, and 12 weeks of HFD (**J**), fasting blood glucose (*n* = 24 to 34) (**K**), and insulin (*n* = 7 to 9) levels (**L**) in *Npr1*^+/−^ and *Npr1*^+/+^ mice fed an SD or HFD. *P ≤ 0.05, ****P < 0.0001, versus *Npr1^+/+^*.

*Npr1*^+/−^ mice had similar body weight gain (Fig. 2A) and body composition (fig. S2, H and I) under SD. *Npr1*^+/−^ mice developed a moderate glucose intolerance (Fig. 2B) despite unchanged insulin sensitivity under SD (Fig. 2C). In the same line, *Npr1*^+/−^ mice exposed to HFD displayed similar body weight gain (Fig. 2D), elevated peak glucose excursion at T15 min (Fig. 2E) and plasma insulin levels (Fig. 2F) during the GTT, reflecting a state of insulin resistance and a discrete deterioration of glucose tolerance. However, when exposed to HFD for 4 weeks (Fig. 2G), 8 weeks (Fig. 2H), and 12 weeks (Fig. 2I), HFD-induced deterioration of insulin sensitivity was further aggravated in *Npr1*^+/−^ versus WT mice (Fig. 2J). Fasting blood glucose (Fig. 2K) and plasma insulin (Fig. 2L) concentrations were increased by HFD in both genotypes, while plasma insulin (Fig. 2L) and HOMA-IR (fig. S2J) tended to increase in *Npr1*^+/−^ mice under HFD. In summary, our data indicate that GCA haploinsufficiency, which phenocopies down-regulation of GCA observed in humans with obesity, impairs glucose homeostasis and promotes insulin resistance and prediabetes.

### GCA haploinsufficiency leads to marginal changes in adipose tissue and liver metabolism

Adipose tissue weight of various depots (fig. S3A) as well as adipocytes area and number in epididymal white adipose tissue (eWAT) (fig. S3, B and C) were similar between *Npr1*^+/−^ and *Npr1*^+/+^ mice. No significant difference in insulin-stimulated glucose uptake was found in various WAT depots [i.e., eWAT, inguinal WAT, and brown adipose tissue (BAT)] (fig. S3D). In parallel, mRNA levels of adipose triglyceride lipase (*Atgl*) and hormone-sensitive lipase (*Lipe*) (fig. S3E), the two main rate-limiting enzymes of lipolysis, as well as the mRNA expression of G0/G1 Switch 2 (*G0s2*) and comparative gene identification 58 (*Cgi58*), two coenzymatic regulators of ATGL activity (fig. S3F), did not differ between *Npr1*^+/−^ and *Npr1*^+/+^ mice. Furthermore, there were also no visible signs of inflammation and macrophage accumulation in the eWAT of *Npr1*^+/−^ mice (fig. S3G). Last, since the ANP/GCA system has been related to BAT activation and thermogenesis (*16*, *17*), we further showed that the expression of *Ppargc1a* and *Ucp1* was similar in both genotypes under HFD where BAT activation and function is suppressed (fig. S3H).

In the same line, insulin resistance in *Npr1*^+/−^ mice appeared independent of major differences in hepatic lipid (fig. S4A) and glycogen content (fig. S4B). We next measured the expression of key limiting enzymes of gluconeogenesis in the liver. PEPCK protein content was similar between *Npr1*^+/−^ and *Npr1*^+/+^ mice (fig. S4C), whereas glucose-6-phosphate (G6Pase) activity was significantly increased (fig. S4D). This phenomenon occurred in the absence of major changes in the expression of genes involved in lipid oxidation (fig. S4E), glucose metabolism (fig. S4F), and de novo lipogenesis (fig. S4G) in the liver of *Npr1*^+/−^ and *Npr1*^+/+^ mice. Collectively, our results suggest that GCA haploinsufficiency does not blunt adipose tissue morphology and glucose uptake, while an elevated activity of a rate-limiting enzyme in gluconeogenesis in the liver is observed.

### GCA haploinsufficiency causes insulin resistance in skeletal muscle

**Fig. 3.**
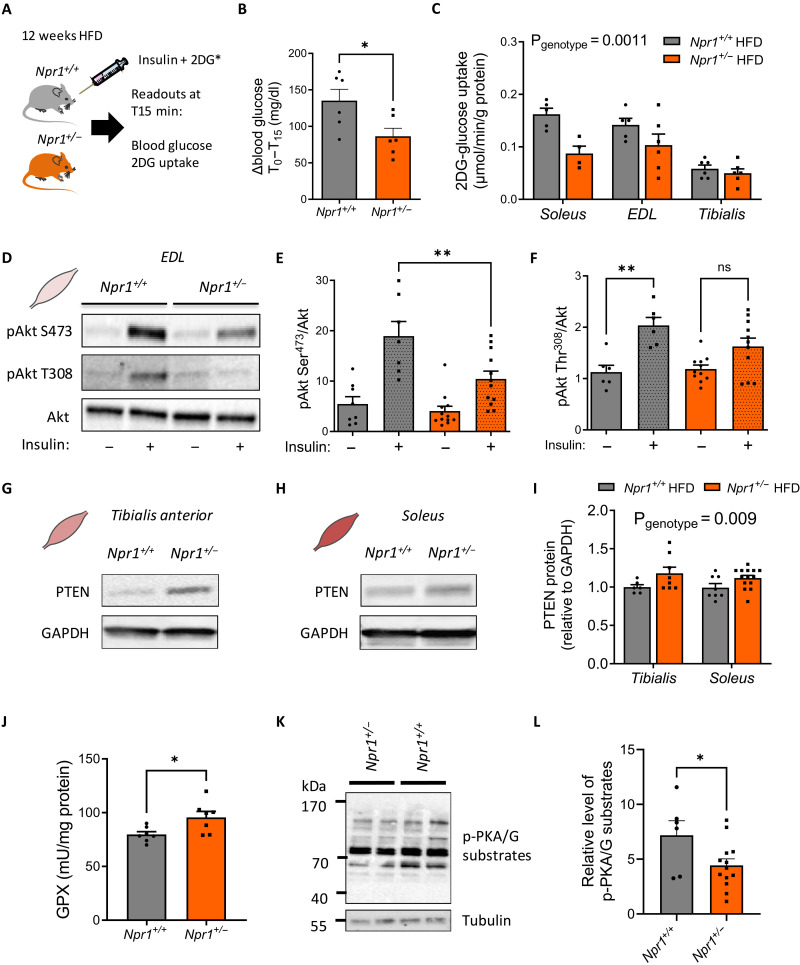
GCA haploinsufficiency causes insulin resistance in skeletal muscle. Insulin and 2-DG bolus injection in *Npr1*^+/+^ and *Npr1*^+/−^ mice (**A**), change in blood glucose levels from t0 to t15 min after injection (**B**), and 2-DG uptake in the soleus, EDL, and tibialis anterior of *Npr1*^+/−^ and *Npr1*^+/+^ HFD-fed male mice after in vivo insulin stimulation (*n* = 6) (**C**). Representative immunoblot (**D**) and relative quantification of Akt-Ser^473^ phosphorylation (**E**) and Akt-Thr^308^ phosphorylation (**F**) to total Akt ratio in the EDL muscle of *Npr1*^+/−^ and *Npr1*^+/+^ HFD-fed mice after ex vivo insulin stimulation (*n* = 8 to 14). Representative immunoblot in tibialis anterior (**G**) and soleus muscle (**H**) and respective quantification of PTEN protein in both muscle of *Npr1*^+/−^ and *Npr1*^+/+^ HFD-fed mice (*n* = 7 to 14) (**I**). GPX activity in the skeletal muscle of *Npr1*^+/−^ and *Npr1*^+/+^ HFD-fed mice (*n* = 7) (**J**). Representative immunoblot (**K**) and quantification of PKA/PKG substrates phosphorylation levels (**L**) in *Npr1*^+/−^ and *Npr1*^+/+^ HFD-fed mice. *P ≤ 0.05, **P < 0.01, versus *Npr1^+/+^*.

Considering the major contribution of skeletal muscle to total glucose disposal (*1*), we next investigated skeletal muscle insulin sensitivity and signaling in HFD-fed *Npr1*^+/−^ mice. The mice were injected with a mixed bolus of insulin and radiolabeled 2-DG as previously described (Fig. 3A) (*18*). We first observed a lower suppression of blood glucose levels in response to insulin in *Npr1*^+/−^ mice (Fig. 3B). This whole-body insulin resistance was associated with a significantly reduced 2-DG uptake in various muscles of *Npr1*^+/−^ mice (Fig. 3C). This observation was consistent with impaired insulin-mediated Akt phosphorylation on Ser^473^ in *Npr1*^+/−^ mice in extensor digitorum longus (EDL) muscle (Fig. 3, D and E). Similarly, insulin-stimulated Akt phosphorylation on Thr^308^ was not significant in *Npr1*^+/−^ mice contrary to WT littermates (Fig. 3, D to F), thus indicating a defect of activation of Akt on both phosphosites.

We further investigated the potential molecular mechanisms behind this muscle insulin–resistant phenotype. Muscle insulin resistance was not associated with changes in inflammatory markers *F4/80*, *Il6*, *Tnfa*, *Mcp1*, *Il1b*, or endoplasmic reticulum stress *Atf4*, *Xbp-1s*, and *Xbp1-us* in *Npr1*^+/−^ mice skeletal muscles (fig. S5, A and B). Previous studies reported that oxidative stress can negatively affect insulin-induced Akt signaling through phosphatase and tensin (PTEN) homolog in skeletal muscles (*19*). We first observed an elevated PTEN protein content in tibialis anterior (Fig. 3, G to I) and soleus muscles (Fig. 3, H and I), thus contributing to down-regulation of Akt-signaling in muscles of *Npr1*^+/−^ mice. This was associated with a trend for lower pyruvate dehydrogenase kinase 1 (PDK1) Ser^241^ phosphorylation level in *Npr1*^+/−^ mice (fig. S5E), a key regulatory kinase upstream of Akt. We next measured the activity of the three main antioxidant enzymes [i.e., superoxide dismutase (SOD), catalase (CAT), and glutathione peroxidase (GPX)]. Both SOD and CAT activities remained unchanged in skeletal muscles of *Npr1*^+/−^ mice compared to WT (fig. S5, C and D). However, GPX activity was significantly increased in *Npr1*^+/−^ mice (Fig. 3J). Consistent with the signaling role of GCA, these molecular changes were associated with a significant decrease of cyclic adenosine monophosphate–dependent protein kinase (PKA)/cyclic guanosine monophosphate (cGMP)–dependent protein kinase (PKG) (RRXS*/T*) substrates phosphorylation level in *Npr1*^+/−^ mice reflecting a reduced GCA signaling in skeletal muscle (Fig. 3, K and L). Together, we here demonstrate that GCA haploinsufficiency induces insulin resistance and defects of insulin signaling potentially through a GPX/PTEN axis in skeletal muscle.

### GCA haploinsufficiency induces mitochondrial dysfunction in skeletal muscle

**Fig. 4.**
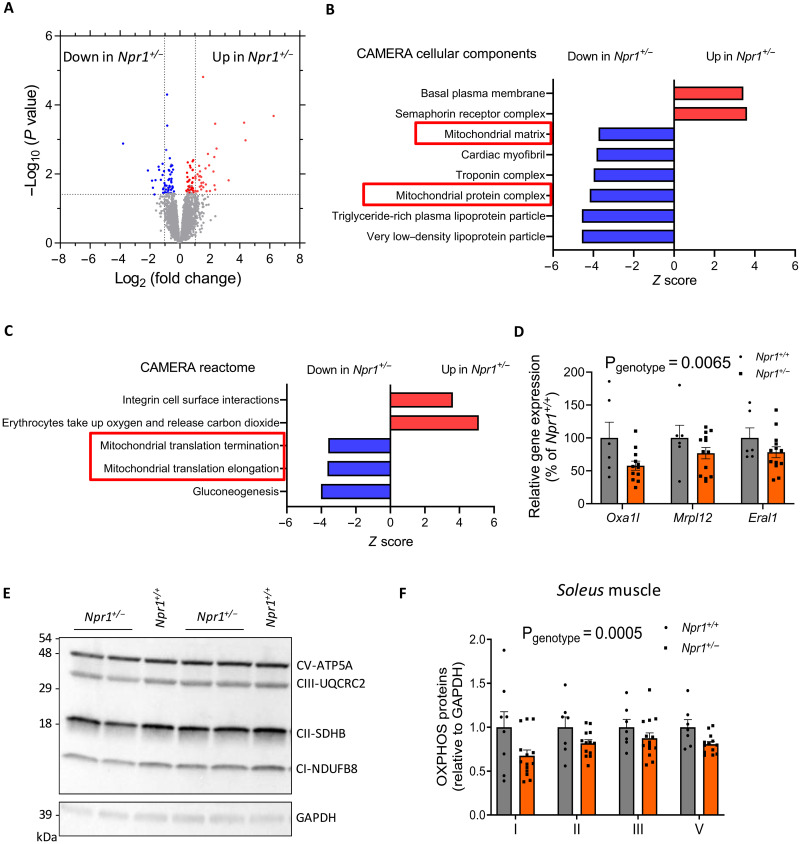
Muscle transcriptomic changes in GCA haploinsufficient obese mice. (**A**) Volcano plot depicting DEGs in skeletal muscle of *Npr1*^+/−^ and *Npr1*^+/+^ HFD-fed male mice. Gene Ontology analysis of the DEGs from (A), showing significantly changed CAMERA cellular components (**B**) and biological pathways (**C**). (**D**) Relative expression of three canonical genes of the mitochondrial translation pathway of *Npr1*^+/−^ and *Npr1*^+/+^ HFD-fed mice. Representative immunoblot (**E**) and relative quantification (**F**) of OXPHOS protein content (*n* = 8 to 14) in the soleus of *Npr1*^+/−^ and *Npr1*^+/+^ mice fed an HFD.

Previous reports suggested that GPX activity is tightly coupled to mitochondrial oxidative capacity and oxidative stress (*20*). Bulk RNA sequencing (RNA-seq) analyses on skeletal muscle revealed 190 differentially expressed genes (DEGs), 108 up-regulated and 82 down-regulated in *Npr1*^+/−^ versus *Npr1*^+/+^ HFD-fed mice (Fig. 4A). Among significant DEGs, gene ontology analyses using CAMERA cellular components (Fig. 4B) and Reactome (Fig. 4C) indicated that various genes related to mitochondrial protein complex, matrix, and translation were down-regulated in *Npr1*^+/−^ mice [false discovery rate (FDR) < 0.05]. We further validated these findings by reverse transcription quantitative polymerase chain reaction (RT-qPCR) and showed that three canonical genes of the mitochondrial translation pathway including *Oxa1l* (OXA1L mitochondrial inner membrane protein), *Mrpl12* (mitochondrial ribosomal protein L12), and *Eral1* (Era-like 12*S* mitochondrial RRNA chaperone 1) were significantly down-regulated in *Npr1*^+/−^ mice (Fig. 4D). These genes cooperate with the mitochondrial ribosome during synthesis of mitochondrial-encoded proteins, thus playing a critical role in oxidative phosphorylation (OXPHOS) complex assembly (*21*). Thus, reduced *Oxa1l* gene expression was associated with a significant down-regulation of various proteins from the OXPHOS pathway encoding mitochondrial respiratory complexes in soleus muscle of *Npr1*^+/−^ mice (Fig. 4, E and F). This observation was further corroborated in other muscle types such as the EDL (fig. S5, F to G) and tibialis anterior of *Npr1*^+/−^ mice (fig. S5, H and I). When investigating mitochondrial mass, there were no obvious changes in subsarcolemal and intermyofibrillar mitochondrial morphology (fig. S6A) and number (fig. S6B) in *Npr1*^+/+^ and *Npr1*^+/−^ mice. Consistently, mitochondrial DNA (fig. S6C) and mass as reflected by citrate synthase activity (fig. S6D) did not differ between genotypes. Muscle fiber cross-sectional area (CSA) remained unaltered both in *Npr1*^+/−^ HFD-fed (fig. S6E) and SD-fed mice (fig. S6F).

**Fig. 5.**
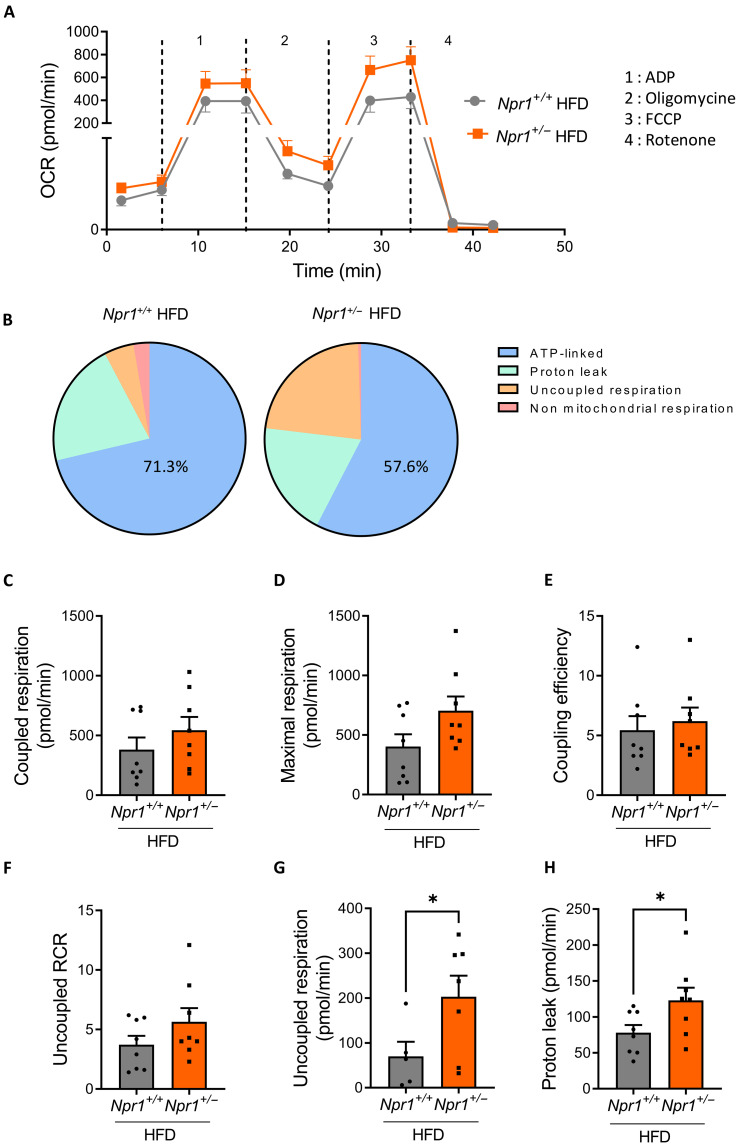
GCA haploinsufficiency induces mitochondrial dysfunction in skeletal muscle. (**A**) OCR in isolated muscle mitochondria and (**B**) relative contribution of various components of mitochondrial respiration of *Npr1*^+/−^ and *Npr1*^+/+^ male mice under fed an HFD (*n* = 8). Coupled respiration (**C**), maximal respiration (**D**), coupling efficiency (**E**), uncoupled RCR (**F**), uncoupled respiration (**G**), and mitochondrial proton leak (**H**) in isolated mitochondria from muscle of *Npr1*^+/−^ and *Npr1*^+/+^ mice under fed an HFD (*n* = 8). *P ≤ 0.05, versus *Npr1^+/+^*.

In addition, mitochondria isolated from muscles of *Npr1*^+/−^ mice presented an elevated oxygen consumption rate (OCR) compared to *Npr1*^+/+^ mice (Fig. 5A). The contribution of adenosine triphosphate (ATP) production rate to total respiration was decreased with a concomitant increase in uncoupled respiration in *Npr1*^+/−^ mice (Fig. 5B). When examining components of mitochondrial respiration, coupled respiration (Fig. 5C), maximal respiration (Fig. 5D), coupling efficiency (Fig. 5E), and uncoupled respiratory control ratio (RCR) (Fig. 5F), did not differ between genotypes. However, uncoupled respiration (Fig. 5G) and mitochondrial proton leak (Fig. 5H) were significantly elevated in *Npr1*^+/−^ mice. Elevated proton leak characterizes an incomplete coupling of oxygen consumption to ATP production and is a hallmark of defective mitochondrial function (*22*, *23*).

Whether mitochondrial dysfunction is a cause or a consequence of diet-induced insulin resistance remains controversial. Some studies indicated that HFD induces mitochondrial abnormalities preceded by insulin resistance (*24*). To assess the specific role of GCA haploinsufficiency on mitochondrial function, we analyzed skeletal muscle mitochondria of *Npr1*^+/−^ mice under SD. In agreement with previous observations under HFD, we showed that OXPHOS protein content was decreased in *Npr1*^+/−^ mice under SD (fig. S7A). As shown previously, we further noticed a significantly increased OCR in skeletal muscle isolated mitochondria and a trend for increased proton leak (fig. S7B) and uncoupled respiration (fig. S7C) in *Npr1*^+/−^ mice. In summary, mitochondrial dysfunction is a primary defect of GCA haploinsufficient mice, thus indicating that mitochondrial defects are concomitant with insulin resistance and caused by GCA deficiency independent of the nutritional state.

### Loss of ANP/GCA signaling impairs endurance capacity and adaptations to exercise training

**Fig. 6.**
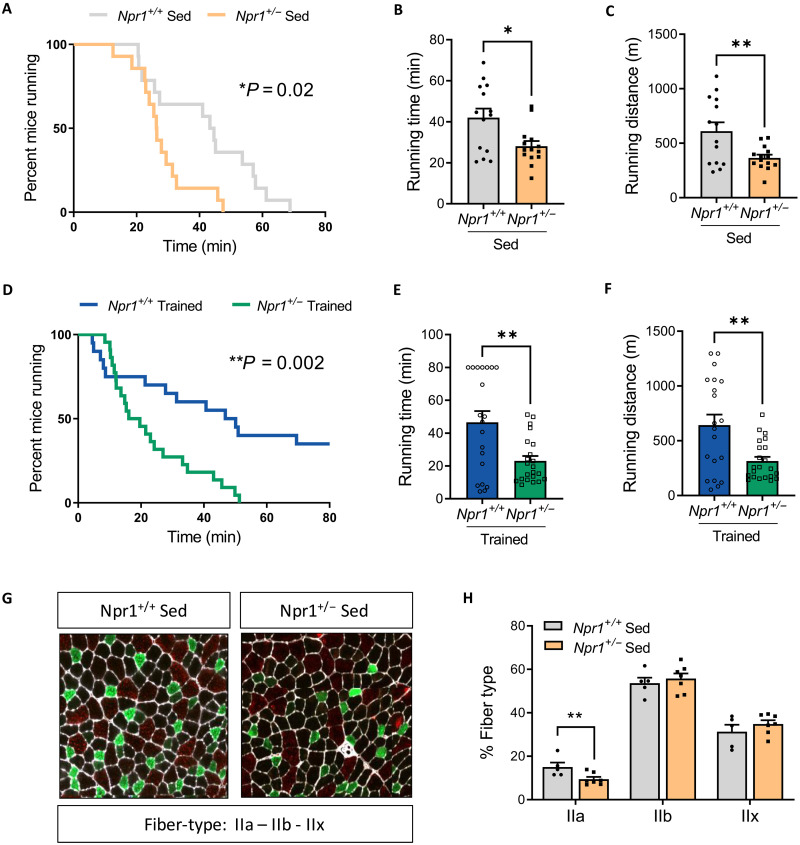
Impaired endurance capacity and response to training in GCA haploinsufficient sedentary mice. (**A**) Percent mice running, (**B**) running time, and (**C**) running distance of *Npr1*^+/−^ and *Npr1*^+/+^ male sedentary (Sed) mice fed an SD during a submaximal endurance test (*n* = 14). Percent mice running (**D**), running time (**E**), and running distance (**F**) of *Npr1*^+/−^ and *Npr1*^+/+^ sedentary (Sed) mice fed an SD during a submaximal endurance test after 4 weeks of endurance training (*n* = 20 to 23). Representative images of tibialis anterior fiber types (**G**) and relative quantification (**H**) in *Npr1*^+/−^ and *Npr1*^+/+^ sedentary mice fed an SD with type IIa fibers (green), type IIb fibers (red), and type IIx fibers (black) (*n* = 5 to 7). *P ≤ 0.05, **P < 0.01, versus *Npr1^+/+^*.

Previous results point toward an aberrant mitochondrial function in the skeletal muscle of *Npr1*^+/−^ mice. Impaired mitochondrial oxidative capacity is associated with poor endurance capacity (*25*). We therefore challenged sedentary (Sed) *Npr1*^+/−^ mice under SD with exercise. Beforehand, we determined individual maximal speed of each mouse. No difference in maximal speed (fig. S7D), running time (fig. S7E), and running distance (fig. S7F) were found between genotypes. However, when submitted to an endurance test at 60% of their maximal speed, *Npr1*^+/−^ mice exhibited a compromised endurance running capacity (Fig. 6A). Consistently, both running time (Fig. 6B) and distance (Fig. 6C) were significantly decreased in *Npr1*^+/−^ mice.

Because muscle oxidative capacity and mitochondrial function can be improved by exercise training (*26*), we next tested whether an exercise training program could partly attenuate the impaired endurance capacity and mitochondrial dysfunction of *Npr1*^+/−^ mice. After 4 weeks of endurance training on a treadmill, *Npr1*^+/+^ mice markedly improved their endurance capacity since 43% of WT mice were still running after 80 min, while 100% had stopped running by 80 min before training (Fig. 6D). *Npr1*^+/−^ mice did not improve their endurance capacity even after training since all mice had stopped running at 51 min (Fig. 6, A and D). Training compliance was good and did not differ between genotypes (fig. S7G), while running time (Fig. 6E) and distance (Fig. 6F) were consistently lower in *Npr1*^+/−^ mice compared to WT mice. OXPHOS protein content again tended to be lower in the skeletal muscle of *Npr1*^+/−^ mice after training (fig. S7H). In addition, OCR in isolated mitochondria from skeletal muscle remained elevated in *Npr1*^+/−^ compared to WT mice (fig. S7I) due to significantly greater proton leak (fig. S7J). Last, these mitochondrial abnormalities and poor endurance capacity were associated with a significant reduction in the number of fast oxidative IIa fibers in the tibialis anterior muscle of *Npr1*^+/−^ mice, while no significant changes in the number of type IIb and type IIx fibers were noted (Fig. 6, G and H). As in *Npr1*^+/−^ mice, we further confirmed impaired mitochondrial OXPHOS complex II protein expression (fig. S8, A and B) and endurance running capacity before (fig. S8C) and following (fig. S8D) a 4-week endurance training program in *Nppa^−/−^* mice. This occurred independently of changes in muscle mass (fig. S8E) and fiber CSA (fig. S8F). Collectively, our data indicate that ANP/GCA deficiency impairs endurance capacity and adaptations to endurance training in mice through loss of mitochondrial oxidative capacity and integrity in skeletal muscle.

### Partial loss of GCA signaling in skeletal muscle causes insulin resistance

**Fig. 7.**
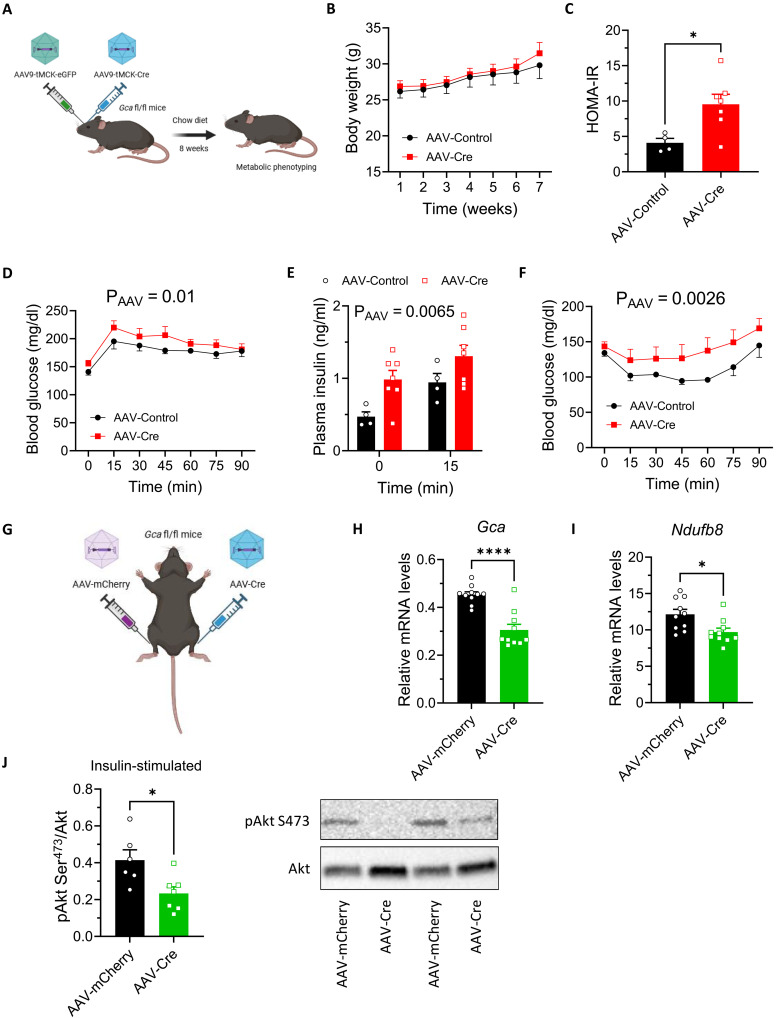
Muscle-specific GCA knockdown impair insulin sensitivity. (**A**) Systemic injection of AAV9-tMCK-eGFP or AAV9-tMCK-iCre in *Gca* fl/fl male mice. Longitudinal body weight (**B**) and HOMA-IR (**C**) in mice injected with AAV-Control (*n* = 4 to 5) and AAV-Cre (*n* = 7). Blood glucose levels during a GTT (**D**), plasma insulin at T0 and T15 of the GTT (**E**), and blood glucose levels during an ITT (**F**) in mice injected with AAV-Control and AAV-Cre. (**G**) Intramuscular injection of AAV1-mCherry or AAV1-Cre in *Gca* fl/fl male mice. *Gca* (**H**) and *Ndufb8* (**I**) relative gene expression, as well as representative blot and quantification of insulin-stimulated Akt-Ser^473^ to Akt phosphorylation level in tibialis anterior muscle (**J**) of mice injected with AAV-mCherry (*n* = 10) or AAV-Cre (*n* = 10). *P ≤ 0.05, ****P < 0.0001, versus AAV-Control or AAV-mCherry.

To delineate the specific role of ANP/GCA signaling in skeletal muscle in the pathogenesis of prediabetes, we developed a muscle-specific GCA knockdown mouse model (MKD-*Gca*) with the use of systemic adeno-associated virus 9 (AAV9)–tMCK-iCre in *Gca* fl/fl mice (Fig. 7A). Twelve-week-old male mice were studied under standard chow diet and HFD. We reached a significant, yet moderate, level of GCA protein down-regulation ranging from 2 to 47% (average 15%) in the tibialis anterior and gastrocnemius muscles of mice injected with AAV-Cre versus AAV-Control (fig. S9A). Down-regulation of GCA was not observed in cardiac muscle (fig. S9B). Consequently, LV mass (fig. S9C), fractional shortening (fig. S9D), and isovolumetric relaxation time (fig. S9E) remained unaltered in mice injected with AAV-Cre. Despite similar body weight gain (Fig. 7B), mice injected with AAV-Cre developed systemic insulin resistance based on HOMA-IR (Fig. 7C), as well as clear signs of prediabetes, as previously observed in *Nppa^−/−^* and *Npr1^+/−^* mice. MKD-*Gca* mice (injected with AAV-Cre) were glucose intolerant (Fig. 7D), had elevated plasma insulin levels at baseline and peak of GTT (Fig. 7E), and reduced insulin sensitivity (Fig. 7F). Despite similar maximal speed (fig. S9G), MKD-*Gca* mice tended to have a lower endurance running capacity when compared to mice injected with AAV-Control (fig. S9H). Eight weeks of HFD strongly deteriorated systemic insulin sensitivity as reflected by the marked elevation of HOMA-IR in these mice yet similarly in mice injected with AAV-Cre and AAV-Control (fig. S9I). Consistently, glucose tolerance (fig. S9J) and insulin sensitivity (fig. S9K) were not further deteriorated in mice injected with AAV-Cre under HFD. As a complementary approach, we next injected *Gca* fl/fl mice into the tibialis anterior muscle with AAV1-mCherry or AAV1-Cre (Fig. 7G). We obtained a significant ~34% down-regulation of *Gca* (Fig. 7H) as well as a down-regulation of *Ndufb8* gene expression levels encoding for a mitochondrial complex I protein (Fig. 7I). Partial loss of GCA signaling was associated with an ~40% reduction of insulin-stimulated Ser^473^-Akt phosphorylation in the tibialis anterior muscle (Fig. 7J).

### GCA correlates with T2D and mitochondrial function–related traits in humans

**Fig. 8.**
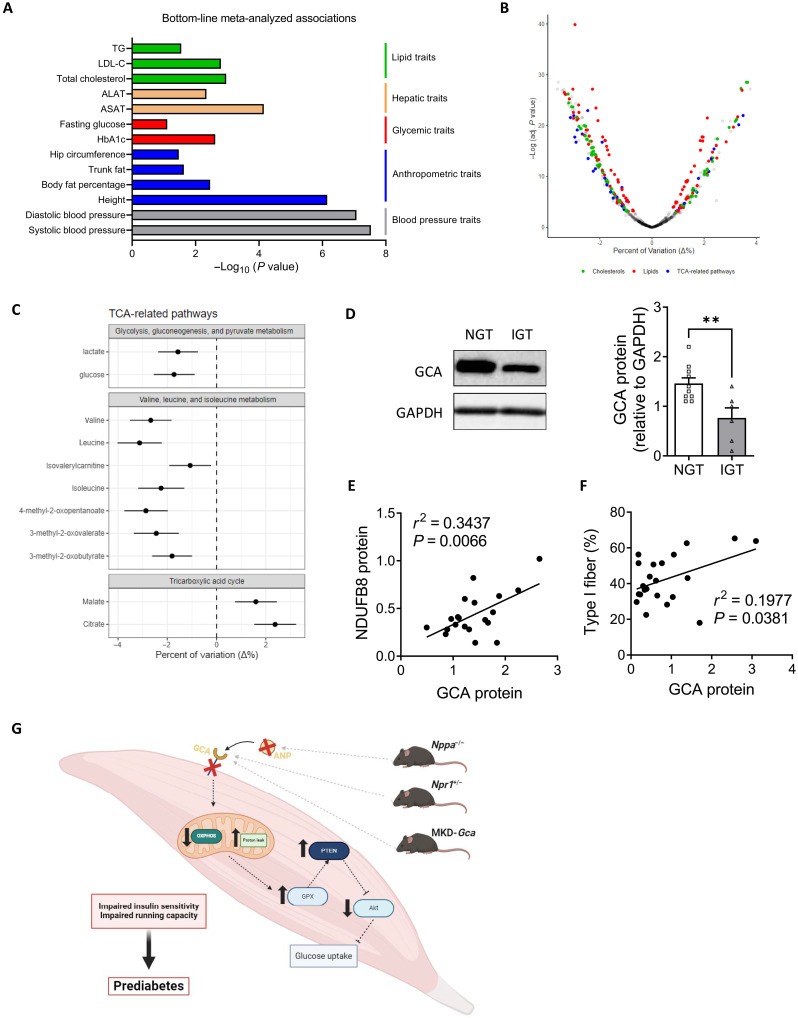
GCA correlates with T2D and muscle mitochondrial oxidative traits in humans. (**A**) Bottom-line meta-analyzed associations between common variants of *NPR1* and metabolic traits extracted from T2DKP. (**B**) Volcano plot depicting correlations between serum MR-proANP and metabolites from various pathways of the NMR, Biocrates, and Metabolon dataset. Each dot represents a metabolite and its color the corresponding class if correlation reached significance. Dots are displayed on the % variation (*x* axis) and the negative logarithm of the adjusted *P* value (*y* axis) and (**C**) Forest plot of significant correlations between serum MR-proANP and various metabolites of the TCA cycle from the KORA cohort of the Metabolon dataset. Dots depict the mean % variation and lines the 95% confidence interval. (**D**) GCA protein level measured in vastus lateralis biopsies of obese individuals with normal glucose tolerance (NGT, *n* = 10) or IGT (*n* = 6), **P < 0.01 versus NGT. Linear regression analysis between muscle GCA protein expression and (**E**) complex I protein-NDUFB8 protein expression (*n* = 20) and (**F**) type 1 fiber content (*n* = 22) in muscle from healthy individuals with a wide-range of clinical characteristics. (**G**) Illustration of findings obtained in the current study.

Our preclinical data in two mouse models prompted us to test whether the results could provide translational value for human patients with T2D. For that purpose, we used the T2D Knowledge Portal (T2DKP; https://t2d.hugeamp.org) database, containing 292 datasets and 326 phenotypic traits, to explore genome-wide association study (GWAS) datasets for the associations of *NPR1* with T2D and related traits. As shown in Fig. 8A, significant associations (*P* < 0.01) between common variants of *NPR1* with systolic and diastolic blood pressure (rs35479618), height and body fat (rs11497739), body mass index (BMI)–adjusted HbA1c and blood glucose (rs11497739), and cholesterol, and TG (rs2282227) were detected. Human Genetic scores (HuGE) were strong for blood pressure and height and moderate for all other variables. We next investigated the correlations between plasma mid-regional proANP (MR-proANP) concentrations and various plasma metabolites measured by metabolomics in the KORA cohort using linear regression models adjusted for age, sex, waist-to-hip ratio, physical activity, glomerular filtration rate, hypertension, and history of myocardial infarction on 1773 individuals (Fig. 8B and fig. S10) (*27*). We observed strong significant negative associations between MR-proANP and biomarkers of T2D such as glucose, lactate, and branched-chain amino acids (BCAAs). We also found positive associations with various intermediate metabolites of the tricarboxylic acid (TCA) cycle such as citrate and malate (Fig. 8C and fig. S10). We also demonstrate a down-regulation of muscle GCA protein level in individuals with impaired glucose tolerance (IGT) (Fig. 8D), while GCA protein expression positively correlates with mitochondrial complex I-NDUFB8 protein levels (Fig. 8E) and type 1 fiber content in healthy individuals (Fig. 8F).

## DISCUSSION

Low biological activity of ANP is clinically tied to obesity, metabolic dysfunction, and increased risk of T2D. However, a direct causal role has not been shown, and the molecular mechanisms involved remain inadequately understood. Here, we show that disruption of ANP/GCA signaling in skeletal muscle is sufficient to cause prediabetes. We further demonstrate that ANP/GCA signaling is required for the maintenance of mitochondrial oxidative capacity in skeletal muscle in vivo in mice and humans (Fig. 8G). This study also provides compelling experimental evidence to prospective cohorts, Mendelian randomization studies, and GWASs in humans that ANP/GCA deficiency is causally involved in the pathogenesis of T2D.

Previous studies reported obesity resistance in NPRC knockout (KO) compared to WT mice (*28*, *29*), thus suggesting a role for natriuretic peptides in the control of energy balance. Here, after 12 weeks of HFD, no differences in body weight and body composition were observed neither between *Npr1*^+/+^ and *Npr1*^+/−^ mice nor between *Nppa*^+/+^ and *Nppa*^−/−^ mice. However, both ANP and GCA deficiency altered insulin sensitivity and glucose homeostasis. We further demonstrate that partial loss of ANP/GCA signaling specifically in skeletal muscle is sufficient to cause systemic insulin resistance and prediabetes. Contrary to global- (*29*) and muscle-specific (*30*) NPRC KO mice, which do not display skeletal muscle metabolic abnormalities or enhanced exercise performance, we observed blunted insulin signaling associated with mitochondrial dysfunction in skeletal muscle of ANP/GCA-deficient mice. Previous studies identified a role of ANP in mitochondrial oxidative capacity. ANP induces mitochondrial biogenesis and increases lipid oxidation in skeletal myocytes in vitro (*15*). Moreover, transgenic mice overexpressing cGMP-dependent protein kinase, the downstream protein of GCA signaling, showed an improved insulin sensitivity and an increased mitochondrial biogenesis associated with the presence of giant mitochondria in skeletal muscles (*31*). In agreement with these studies, we here show that *Npr1*^+/−^ mice have dysfunctional mitochondria. First, muscle transcriptome analyses by RNA-seq revealed a down-regulation of several gene sets related to mitochondrial protein complex and formation such as *Oxa1l* (OXA1L mitochondrial inner membrane protein), which encodes an evolutionarily conserved protein that is localized to the inner mitochondrial membrane and required for mitochondrial complexes assembly (*32*). Consistent with muscle transcriptome changes, GCA haploinsufficient mice exhibited increased mitochondrial proton leak, a hallmark of defective mitochondria. The decrease of proton gradient across the inner mitochondrial membrane by proton leak reduces mitochondrial efficiency and ATP production rate and induces energy dissipation (*23*). The increased OCR observed in isolated muscle mitochondria of *Npr1*^+/−^ mice is likely a compensatory mechanism to sustain sufficient ATP production. A similar phenotype is observed in mice with muscle-specific overexpression of uncoupling protein 3, which enhances mitochondrial proton leak and oxygen consumption in isolated mitochondria despite a 42% reduced efficiency of muscle mitochondrial oxidative phosphorylation in vivo (*22*). It is well-known that the electron transport chain is the primary source of reactive oxygen species and that oxidative stress may be induced by defective mitochondria (*23*). Thus, we investigated antioxidant enzyme activities and unexpectedly found an increased GPX activity in *Npr1*^+/−^ mice. A prior study on *Gpx* KO mice revealed that *Gpx1* deficiency enhances insulin signaling in vivo by the inhibition of PTEN, a negative modulator of the PI3K/Akt pathway, to attenuate the development of insulin resistance (*19*). In line with these findings, a previous study indicated that *Gpx1* overexpression induces insulin resistance in mice (*33*). In addition, it has been shown that GPX can be induced in response to oxidative stress (*20*). Consequently, we propose the following model in *Npr1*^+/−^ mice: Defective leaky mitochondria increase GPX activity, which in turn increases PTEN protein level and impairs insulin signaling in skeletal muscle.

As the causal effect of defective oxidative capacity in the development of insulin resistance remains controversial, we aimed to explore mitochondrial function in *Npr1*^+/−^ mice before the onset of insulin resistance. Under SD and sedentary conditions, *Npr1*^+/−^ mice already have deficient muscle mitochondria, with a trend toward increased proton leak and uncoupled respiration associated with reduced OXPHOS protein content. Combined, this molecular signature is detrimental for exercise performance and adaptation to training (*34*). Thus, GCA haploinsufficient mice exhibit low-endurance capacity when subjected to forced exercise on a treadmill. A similar phenotype is recapitulated in global ANP KO mice and in mice with muscle-specific GCA knockdown. This phenotype is observed under standard sedentary and nutritional conditions in the absence of obesity. The low-endurance running capacity could also result from altered skeletal muscle fiber composition in *Npr1*^+/−^ mice displaying a lower type IIa oxidative fiber content. This is in agreement with a recent study showing an increase of *Myh7* expression, the major myosin heavy chain isoform of oxidative fibers (i.e., type I fibers) and a decrease of *Myh1* expression, the major isoform of type IIx fibers, in skeletal muscles of NPRC KO mice (*28*). These results reveal that mitochondrial dysfunction is already present under baseline sedentary and nutritional conditions and induces insulin resistance when *Npr1*^+/−^ mice are challenged by an HFD. Despite potential qualitative changes in fiber-type composition, no functional impairments in terms of fiber size, muscle mass, and strength were observed in any of the ANP/GCA-deficient mouse models investigated.

Since global ANP/GCA deficiency may trigger various confounding effects on phenotype interpretation, we next investigated the causal role of muscle-specific GCA knockdown by the use of AAV delivery to *Gca* fl/fl mice. Our data indicate that partial loss of ANP/GCA signaling specifically in skeletal muscle is sufficient to cause systemic insulin resistance and prediabetes under standard nutritional conditions. This phenotype was not further aggravated under HFD. These mice also displayed a trend for reduced endurance running capacity associated with lower expression of the mitochondrial complex I protein NDUFB8. The level of knockdown reached was rather moderate because of a limited load of AAV delivered, possibly implying that higher knockdown would have produced more robust differences. We cannot completely rule out that additional factors such as impaired muscle capillary density and/or blood flow due to global loss of ANP/GCA signaling may have contributed to impaired endurance running capacity.

We further validated the clinical translation of our findings in various human studies and cohorts. We confirmed that *NPR1* gene variants were associated with various T2D traits such as HbA1c and fasting glucose in a large database of GWAS studies from the T2DKP. MR-proANP was recently reported as a significant predictor of incident T2D in the large prospective KORA cohort (*27*). We here further show that plasma MR-proANP levels negatively correlate with glucose and BCAA (*27*). BCAA have been previously reported as a biomarker of T2D (*35*). We also observed that MR-proANP positively correlates with intermediate metabolites of the TCA cycle, reflecting an association between ANP and mitochondrial oxidative metabolism in vivo in humans. In agreement with our findings in mice partially deficient for GCA, we also found a positive association between GCA protein content and molecular markers of oxidative capacity (mitochondrial complex I protein-NDUFB8 and fiber-type 1 content) in human skeletal muscle. We finally found a nearly 50% reduction of GCA protein level in the skeletal muscle of obese individuals with prediabetes (IGT) versus normal glucose tolerance (NGT). This observation is consistent with previous reports demonstrating a down-regulation of GCA gene and protein expression in adipose tissue and skeletal muscle of people living with obesity when compared to lean healthy controls (*10*, *12*, *14*).

Collectively, these results provide compelling evidence for a causal link between ANP/GCA deficiency in skeletal muscle and the development of insulin resistance and poor endurance capacity in mice and humans. These translational data highlight the critical importance of ANP/GCA signaling for the maintenance of skeletal muscle insulin sensitivity and oxidative capacity in vivo. Thus, therapeutic strategies aimed at maintaining or improving ANP/GCA signaling in skeletal muscle may convey various health benefits and help preventing and treating prediabetes.

## MATERIALS AND METHODS

### Animals

Twelve-week-old male *Nppa^−/−^* and *Npr1^+/−^* male mice (B6.129P2-*Nppa*^tm1Unc^/J) and B6.129-Npr1^tm1Gar^/J mice were backcrossed to C57BL/6J mice for at least 10 generations), and their littermate controls *Nppa^+/+^* and *Npr1^+/+^*, respectively, were used. Twelve-week-old male *Gca* fl/fl mice (*36*) were used for systemic and intramuscular AAV injections to produce a muscle-specific GCA knockdown (MKD-*Gca*). Mice received either retro-orbital injections of AAV9-tMCK-eGFP-WPRE or AAV9-tMCK-iCre-WPRE (Vector Biolabs, Malvern, USA) at 2 × 10^12^ vg/kg, or intramuscular injection of AAV1-mCherry or AAV1-Cre at 5 × 10^10^ vg in the tibialis anterior muscle under anesthesia. Mice were fed with an SD (Sniff) or high HFD (60% kcal lipids) up to 12 weeks and were housed in a pathogen-free barrier facility (12-hour light/dark cycle) with ad libitum access to water and food in standard animal care facility rooms at 21°C. The mice were weighted weekly, and body composition was assessed by the use of an EchoMRI. At the end of the protocol, the mice were euthanized and blood was collected into EDTA tubes containing protease inhibitors. The mice injected with AAV were euthanized 8 to 12 weeks after injection. Organs and tissues were rapidly excised and either snap frozen in liquid nitrogen before being stored at −80°C or processed for histology or ex vivo testing. All experimental procedures were approved by our institutional animal care and use committee CEEA-122 of UMS006 CREFRE (protocol # 2016122311033178) and performed according to INSERM guidelines for the care and use of laboratory animals.

### Blood analyses

Mouse plasma ANP and BNP were measured with mouse ANP enzyme-linked immunosorbent assay (ELISA) kit (Cusabio) and mouse brain natriuretic peptide ELISA kit (Cusabio), respectively, following the manufacturer’s instructions. Plasma insulin was measured using an ultrasensitive ELISA kit (ALPCO Diagnostics, Salem, New Hampshire).

### Glucose and insulin tolerance tests

After a 2-h fast (*37*), the mice were injected intraperitoneally with a bolus of insulin (NovoRapid, 100 IU/ml), and blood glucose levels were monitored from the tip of the tail vein with a glucometer (Accucheck; Roche, Meylan, France) at 0, 15, 30, 45, and 60 min after injection. Mice under SD were injected with 0.5 U/kg and mice under HFD with 0.75 U/kg of insulin, respectively. For GTT, mice under SD and HFD were gavaged with 1.5 g/kg of glucose (Sigma-Aldrich, Saint-Quentin Fallavier, France).

### Ex vivo insulin stimulation

Just after removal, EDL muscles were incubated 20 min at 37°C in a KRHA buffer [1.18 M NaCl, 10 mM CaCl_2_, 10 mM MgSO_4_, 10 mM KH_2_PO_4_, 60 mM KCl, 0.2% bovine serum albumin (BSA), 20 mM Hepes, and 2 mM sodium pyruvate] supplemented or not with insulin (100 mM). Muscles were then dried and frozen at −80°C.

### Tissue-specific 2-DG uptake in vivo

To determine 2-DG uptake in muscle and adipose tissue, a bolus injection of 2-[1,2-^3^H(N)]deoxy-d-glucose (PerkinElmer, Boston, Massachusetts) (0.4 μCi/g body weight) and insulin (3 mU/g body weight) was injected intraperitoneal in mice as previously described (*18*). Then, 15 min after injection, blood glucose was measured, the mice were euthanized, and the tissues were excised and quickly frozen at −80°C until further processing. The metabolic clearance rate of glucose was calculated as follows: Rg = [(2-[^3^H]DGP_muscle_/2-[^3^H]DG SA)]*100000)/15, where 2-[^3^H]DGP_muscle_ is the 2-[^3^H]DGP radioactivity counts in muscle (dpm.g^−1^) and 2-[^3^H]DG SA is the specific activity of glucose (dpm.μmol^−1^). Rg was expressed in μmol · 100 g tissue · min^−1^ as previously described (*38*).

### Acclimation to treadmill and maximal running capacity test

Mice were acclimated to the treadmill (TSE Systems) four consecutive days by running for 2 min at a speed of 0.10 m/s, then 3 min at a speed of 0.10 ms/s, and, finally, 7 min at a speed of 0.20 m/s at 0° incline during the week prior the maximal running capacity test. The maximal running capacity test to determine maximal speed was performed at 0.15 m/s and was increased by 0.05 m/s every 2 min until exhaustion.

### Endurance exercise test

For the endurance exercise test, after a warm up of 2 min at a speed of 0.10 m/s, the mice ran for 80 min at 60% of maximal capacity previously determined for each mouse. At exhaustion, the mice were removed from the test and running time to exhaustion, distance covered, and average running speed were measured.

### Endurance training

The endurance training group ran for 30 min per day, 5 days a week for 4 weeks. On the first week of training, the running speed was fixed at 60% of maximal capacity for each mice and was increased at 65, 70, and 75% for weeks 2, 3, and 4, respectively, of training.

### CAT, SOD, and GPX activity

Antioxidative activities including SOD, CAT, and GPX activities were measured in skeletal muscle as previously described (*38*).

### Histology

Adipose tissues and liver were fixed with 4% paraformaldehyde in phosphate-buffered saline (PBS), dehydrated, embedded in paraffin, and cut into 7-μm sections. The sections were stained with hematoxylin and eosin using standard protocols (*17*).

### Transmission electron microscopy

Plantaris muscles were fixed with 4% glutaraldehyde and 0.3 M sodium cacodylate. Small pieces of 1 to 2 mm^3^ were prepared using a scalpel and stored in fixation buffer at 4°C until analysis. To obtain a valid representation of the whole muscle, two micrographs (one from the subsarcolemal region and one from the adjacent intermyofibrillar region) in three separate muscle fibers of the gastrocnemius muscle from each genotype were acquired for a total of six micrographs per mouse. Mitochondrial content was determined using the point-counting stereological analysis methodology with ImageJ software (ImageJ 1.41, National Institutes of Health, Bethesda, MD). Each micrograph was counted and then recounted in a double-blind fashion.

### Fiber typing

Gastrocnemius muscles were mounted in OCT freezing medium, snap-frozen in liquid N2-cooled 2-methylbutane, and stored at −80°C for cryo-sectioning. Tissues were cut into 10-μm section using Leica CM1850 cryostat. For immunostaining, the tissues were treated with mouse-on-mouse reagents (M.O.Mkit, Vector Laboratories) to block endogenous Fc receptor–binding sites. The sections were then labeled with a mixture of mouse monoclonal antibodies BA-D5 (anti-MYH7, fiber type I), SC-71 (anti-MYH2, fiber type IIa), and BF-F3 (anti-MYH4, fiber type IIb) (hybridoma supernates, Developmental Studies Hybridoma Bank) supplemented with rabbit polyclonal anti-laminin antibody (2 μg/ml, Sigma-Aldrich) overnight at 4°C. The sections were then incubated with isotype-specific Alexa Fluor (AF)–conjugated secondary antibodies (Jackson immune Laboratory): anti-mouse Blue-AF 405 immunoglobulin G2b (IgG2b, #115-475-207), anti-mouse Green-AF 488 IgG1 (115-545-205), anti-mouse Red-AF 546 IgM (#115-295-020), and anti-rabbit Far Red-AF 647 (#711-175-153) to mark type I, IIa, IIb fibers, and laminin, respectively. The sections were mounted using Fluoromount G medium (Interchim, #FP-483331). All images were obtained using a Nikon Eclipse Ti-E inverted epifluorescent microscope equipped with a motorized stage. The images were acquired in NIS-Elements software (Nikon) with a 10×/0.3–numerical aperture PlanFluor objective to form a single image of the entire muscle cross section (approximately 20 to 30 mm^2^) used for analysis. The analyses were performed on the entire muscle cryosection using Open-CSAM tool in the FIJI/ImageJ software (*39*).

### Mitochondria isolation from skeletal muscle

Freshly dissected red part of gastrocnemius was placed in ice-cold buffer 1 for mitochondrial isolation (IBM1) containing 67 mM of sucrose, 50 mM tris/HCl, 50 mM KCl, 10 mM EDTA/tris, and 0.2% BSA (pH 7.4) (*40*). The muscle was minced into very small pieces. The tissue was transferred to a cell strainer, rinsed with IBM1, and placed in IMB1/0.05% trypsin for digestion for 30 min in ice. The sample was then centrifuged at 300*g* for 3 min at 4°C, the supernatant was removed, and the pellet was resuspended in IBM1. The sample was homogenized using a Potter Ehlvejhem glass/Teflon homogenizer (Thomas Scientific, Swedesboro, NJ) and centrifuged at 700*g* for 10 min at 4°C. The supernatant was transferred to a polypropylene tube and was centrifuged at 8000*g* for 10 min at 4°C. The supernatant was carefully removed and the pellet suspended in buffer 2 for mitochondrial isolation (IBM2) containing 200 mM mannitol, 70 mM sucrose, 5 mM EGTA/tris, and 10 mM tris/HCl (pH 7.4) and then centrifuged again at 8000*g* for 10 min at 4°C. Last, the supernatant was carefully discarded, and the pellet was resuspended in 100 μl of IBM2. Protein concentration was determined using the bicinchoninic acid (BCA) Pierce assay.

### Seahorse

XF24 instrument was equilibrated at 37°C overnight. Five micrograms of mouse skeletal muscle mitochondria was plated in each well of the XF24 v7 plate in a volume of 50 μl containing 1 × MAS [70 mM sucrose, 220 mM mannitol, 10 mM KH_2_PO_4_, 5 mM MgCl_2_, 1 mM EGTA, 2 mM Hepes, and 0.2% BSA (pH 7.2)] with 10 mM pyruvate and 5 mM malate. The XF plate containing mitochondria with substrate was centrifuged for 20 min at 2000 rpm at 4°C. After centrifugation, 450 μl of substrate containing 1 × MAS with pyruvate and malate was added to each well, and the plate was incubated in a 37°C non-CO_2_ incubator for 10 min. While the plate was being centrifuged, the XF cartridge was prepared for injections for ports A, B, C, and D. Adenosine diphosphate (ADP, 40 mM), oligomycin (25 μM), carbonyl cyanide *p*-trifluoromethoxyphenylhydrazone (FCCP, 40 μM), and rotenone (40 μM) were loaded into ports A, B, C, and D, respectively. The final concentrations were 4 mM ADP, 2.5 μM oligomycin, 4 mM FCCP, and 4 μM antimycin A. The cartridge was calibrated by the XF machine, and after calibration, the XF plate with mitochondria attached to the bottom was introduced into the machine for the run.

### RNA extraction and real-time qPCR

Total RNA from tissue was isolated using Qiagen RNeasy kit (Qiagen, GmbH Hilden, Germany) following the manufacturer’s protocol. The quantity of the RNA was determined on a Nanodrop ND-1000 (Thermo Fisher Scientific, Rockford, IL, USA). RT-PCR was performed using the Multiscribe reverse transcriptase method (Applied Biosystems, Foster City, CA). qRT-PCR was performed in duplicate using the ViiA 7 real-time PCR system (Applied Biosystems). All expression data were normalized by the 2^(-ΔCt)^ method using *TBP* as housekeeping gene. Primer sequences are listed in table S1.

### LIME study

Muscle vastus lateralis biopsies were obtained from 22 sedentary men aged between 34 and 53 years participating in the LIME study (*41*). Of these, 6 were normal weight controls (BMI ≤ 25 kg/m^2^), 10 were obese (BMI > 30 kg/m^2^) with NGT, and 6 were obese with IGT. Sedentary lifestyle was defined by the absence of participation in regular leisure time or intense physical activity over the three previous months or longer.

### RNA-seq

Bulk mRNA sequencing was performed by the Single-Cell Omics platform at the Novo Nordisk Foundation Center for Basic Metabolic Research. Libraries were prepared from total RNA. Libraries were prepared using the Illumina TruSeq Stranded mRNA protocol (Illumina) as recommended by the manufacturer. In brief, poly-A–containing mRNAs were purified by poly-T attached magnetic beads and fragmented, and cDNA was synthesized using SuperScript III Reverse Transcriptase (Thermo Fisher Scientific). cDNA was adenylated to prime for adapter ligation, and after a clean-up using AMPure beads (Beckman Coulter), DNA fragments were amplified using PCR followed by a final clean-up. Libraries were quality-controlled using a TapeStation instrument (Agilent Technologies) and subjected to 52–base pair paired-end sequencing on a NovaSeq 6000 (Illumina). Preprocessing of sequencing data was performed by the Single-Cell Omics platform at the Novo Nordisk Foundation Center for Basic Metabolic Research. In short, sample-demultiplexed FASTQ files were generated from BCL files using the bcl2fastq software (v. 2.20.0.422), and alignments were then generated by mapping the reads to the GRCm38 genome using STAR [v. 2.7.2b, (*42*)], with the GENCODE version M24 gene model (*43*). Gene counts were quantified using featureCounts v1.6.2 (*42*) with the same gene model as when mapping. Fragments were only counted where both ends of the fragment were aligned. Differential gene expression and gene ontology analyses were similarly performed by the Single-Cell Omics platform at the Novo Nordisk Foundation Center for Basic Metabolic Research. Here, the muscle tissue was analyzed using the EdgeR [v. 3.38.0, (*44*)]. R packages compared the two genotypes while correcting for cage and body weight effects. Specifically, the differential gene expression analyses were performed using the glmQLFit-function with robust = TRUE, while gene ontology analyses were performed using the Camera test (*45*), only investigating terms with between 5 and 500 expressed genes. Bulk RNA-seq data have been deposited in Gene Expression Omnibus under the accession number GSE245048.

### KORA study

The KORA F4 cohort is a follow-up study from the previous KORA S4 survey, which enrolled inhabitants of German nationality between the ages of 25 and 74 years old from the region of Augsburg, South Germany. Data from 4227 participants were collected between 2006 and 2008. The study design, standardized sampling method, and data collection have been described in detail elsewhere (*46*–*48*). The KORA cohort ethical approval was granted by the ethics committee of the Bavarian Medical Association (REC reference number: F4: #06068), and all were carried out in accordance with the principles of the Declaration of Helsinki. This covers consent for the use of biological material, including genetics. All research participants have signed informed consent before taking part in any research activities. The KORA data protection procedures were approved by the responsible data protection officer of the Helmholtz Zentrum München.

### NMR serum metabolomics

The participants were fasted when blood samples were collected. Metabolite detection and quantification were performed on a high-throughput nuclear magnetic resonance (NMR) spectroscopy–based platform (Nightingale Ltd., Helsinki, Finland) (*49*, *50*). A total of 228 serum metabolic measures were assessed, and after data quality control, 226 remained: 147 directly measured, mostly given in concentration units, and 79 derived ratios, mostly given in percentage, such as the ratios of specific types of lipids to total lipids in lipoprotein subclasses. Further details on sample preparation and the metabolic measure data can be found elsewhere (*51*).

### Biocrates

Targeted metabolomics measurements have been performed at the Metabolomics Platform of Helmholtz Munich. Serum samples were measured with the AbsoluteIDQTM p150 Kit (Biocrates Life Sciences AG, Innsbruck, Austria). Serum samples have been analyzed using flow injection–electrospray ionization–tandem mass spectrometry (FIA-ESI-MS/MS). Details of the assay procedure as well as nomenclature was previously reported (*52*).

### Metabolon

Serum metabolites from KORA were quantified at Metabolon Inc. (Durham, NC) using a nontargeted metabolomics gas and liquid chromatography coupled to mass spectrometry (GC/MS and LC/MS, respectively) approach. Details of the methods applied for quantification and identification of metabolites were reported previously (*53*, *54*).

### Analysis

Data analysis and visualization were performed using R (4.3.1) with the Rstudio (2023.06.0 + 421) interface and packages tidyverse (2.0.0), broom (1.0.5), stats (4.3.1), and jtools (2.2.2). To achieve normal distribution, metabolite signals and MR-proANP concentrations were log-transformed. Afterward, outlier detection was performed where values outside four standard deviations from the mean were removed. Data were then scaled by subtracting the values of each metabolite from its mean and dividing by its standard deviation. Metabolites with more than 20% and samples with more than 10% missing values were excluded from the data. Association between MR-proANP concentrations and metabolites was assessed using linear regression models adjusted for age, sex, waist-hip ratio, physical activity, glomerular filtration rate, hypertension, and history of myocardial infarction. On the basis of the regression coefficient β representing the increase or decrease of the respective metabolite to one unit increase or decrease of the MR-proANP concentration, the percent of the variation was calculated as follows: (*e*^β^−1) * 100. Then, *P* values were adjusted for multiple testing by controlling the FDR according to the method of Benjamini and Hochberg. A *P* value below 0.05 was considered statistically significant. Metabolites were, if possible, added to pathways according to the human metabolome database. Reproducibility was checked by comparing the results between the NMR, Biocrates, and Metabolon datasets (fig. S10).

### Western blot

Proteins were extracted from tissues using radioimmunoprecipitation assay buffer and protease inhibitor cocktail (Sigma-Aldrich). Tissues homogenates were centrifuged twice for 20 min at 12,700 rpm, and supernatants were quantified with BCA Pierce kit (Thermo Fisher Scientific). Equal amount of proteins were run on a 4 to 20% SDS–polyacrylamide gel electrophoresis (Bio-Rad), transferred onto nitrocellulose membrane (Bio-Rad), and incubated overnight at 4°C with primary antibodies as follows: rabbit glyceraldehyde-3-phosphate dehydrogenase (1:1000, CST, #2118), rabbit GCA (1:1000, Abcam, ab154266), rabbit PEPCK (1:7000, Santa Cruz Biotechnology, #32879), pS473 AKT (1:1000, CST, #4060), pT308 AKT (1:1000, CST, #13038), AKT (1:1000, CST, #4691), pS241 PDK1 (1:1000, CST, #3061), PDK1 (1:1000, CST, #3062), PTEN (1:1000, CST, #9552), p-PKA substrate (RRXS*/T*) (1:1000, CST, #9624), OXPHOS (1:1000, Abcam, ab110411), and rabbit β-actin (1:10000, CST, #4970). Subsequently, immuno-reactive proteins were blotted with anti-rabbit or goat horseradish peroxidase–labeled secondary antibodies for 1 hour at room temperature and revealed by enhanced chemiluminescence reagent (SuperSignal West Femto, Thermo Fisher Scientific), visualized using ChemiDoc MP Imaging System, and data analyzed using the ImageLab 4.2 version software (Bio-Rad Laboratories, Hercules, USA).

### Echocardiography

Echocardiography was carried out with a Vivid7 echograph (GE Healthcare) and a 14-MHz transducer (i13L, GE) on lightly anesthetized (1% isoflurane in air) mice placed on a heating pad, as previously described (*55*). LV walls and cavity dimensions were obtained from parasternal short axis view at mid-ventricular level during Time Movement mode acquisition. LV mass was estimated by a spherical approximation. LV ejection fraction was measured from parasternal long axis view by delineating LV chamber area in diastole and systole. The operator was blind from mice genotype.

### G6Pase activity

Frozen tissues were homogenized using Fast Prep in 10 mM Hepes and 0.25 M sucrose (pH 7.4) (9 vol./g tissue). G6Pase activity was assayed in homogenates for 10 min at 30°C at pH 7.3 in the presence of a saturating G6Pase concentration (20 mM) (*56*).

### Liver glycogen content

Liver samples were weighed and homogenized in acetate buffer (0,2 M, pH 4.8). After centrifuging the samples at 12,000*g* for 10 min, the supernatant was transferred into clean tubes and divided in two aliquots. An aliquot of each homogenate was mixed with α-amyloglucosidase (Sigma-Aldrich) and incubated at 55°C for 15 min. The other one was mixed with water and incubated at 4°C for 15 min. Glucose content was measured as previously described (*37*). The samples were analyzed in duplicate and the results determined as microgram glycogen per milligram tissue.

### Grip strength

For grip strength assessment, each mouse was allowed to grab a grid, with forelimbs and hindlimbs attached to a force transducer (Bioseb) as it was pulled away by the tail horizontally. Three independent trials were averaged for each mouse as previously described (*57*).

### Muscle fiber CSA

Muscles were quickly dissected, mounted in 9% Tragacanth gum (Sigma-Aldrich, G1128), frozen in liquid nitrogen–cooled isopentane, and kept at −80°C. Ten-micromolar cryosections were blocked with mouse on mouse (M.O.MTM) blocking reagent (MKB-2213, Vector Laboratories) before overnight incubation at 4°C with anti-laminin (L9393, Sigma-Aldrich) primary antibody in Dulbecco’s PBS (DPBS) buffer supplemented with 0.5% BSA (A7030, Sigma-Aldrich). The next day, the slides were washed in DPBS and stained with anti-rabbit Far Red-Alexa Fluor 647 (711-175-152, Jackson ImmunoResearch) secondary antibody, in DPBS buffer supplemented with 0.5% BSA, for 1 hour at 37°C. After washing in DPBS, the slides were mounted in Fluoromount G medium (FP-483331, Interchim, Montluçon, France). Whole-muscle section images were acquired at a 10× magnification with a wide-field fluorescence video-microscope (Video Microscope Cell Observer, ZEISS, Oberkochen, Germany). Mosaics were then stitched (Zen 2.3 lite, Zeiss). For each sample, altered fibers were manually removed. Segmentation of all muscle fibers from a cryosection was performed using Cellpose (*58*, *59*). Mean fiber CSA was obtained using a self-developed Python script using all muscle fibers from each section.

### Statistical analyses

All statistical analyses were performed using GraphPad Prism 9.5.0 for Windows (GraphPad Software Inc., San Diego, CA). Normal distribution and homogeneity of variance of the data were tested using Shapiro-Wilk and *F* tests, respectively. Student’s *t* tests, Mann-Whitney test, and two-way analysis of variance (ANOVA) followed by Bonferonni’s post hoc tests were applied when appropriate. All values in figures are presented as means ± SEM. Statistical significance was set at *P* < 0.05.

## Supplementary Material

20241009-1

## Notes

**Correction (10 October 2024):** Due to a production error, the incorrect figure file for Fig. 2 was included in both the online and PDF versions of the original article. The figure has been updated to the correct file online and in the PDF.
