# Supplementary material for: Loss of atrial natriuretic peptide signaling causes insulin resistance, mitochondrial dysfunction, and low endurance capacity
Source: Sci Adv. 2024 Oct 9;10(41):eadl4374. doi: 10.1126/sciadv.adl4374 (PMC11463261; doi:10.1126/sciadv.adl4374)
Supplement: Supplementary file 2 — Tables S1 and S2 Figs. S1 to S10 [file sciadv.adl4374_sm.pdf]

Science Advances
AAAS

Supplementary Materials for

Loss of atrial natriuretic peptide signaling causes insulin resistance, mitochondrial dysfunction, and low endurance capacity

Deborah Carper *et al.*

Corresponding author: Cedric Moro, cedric.moro@inserm.fr

Sci. Adv. **10**, eadl4374 (2024)
DOI: 10.1126/sciadv.adl4374

This PDF file includes:

Tables S1 and S2
Figs. S1 to S10

Supplementary Table 1. Primers and probes list for qPCR analyses using Taqman chemistry

Gene symbol	Taqman Probe
Cpt1b	Mm00487200_m1
Npr1	Mm00435324_m1
Npr3	Mm00435329_m1
Ppargc1a	Mm01208835_m1
Ucp1	Mm01244861_m1
18s	Hs99999901_s1
F4/80	Mm00802530_m1
Il6	Mm00446190_m1
Tnfα	Mm00443258_m1
Tfam	Mm00447485-m1
Tgfβ	Mm01178819_m1
G0s2	Mm00484537-g1
Cgi58	Mm00470731_m1
Lipe	Mm00495353_m1
Gys1	Mm_00472712-m1
Glut2	Mm00446229-m1
Pepck	Mm00440636_m1
Srebp1	Mm01138344_m1

Supplementary Table 2. Primer sequences list for qPCR analyses using SYBR chemistry

**Gene symbol**	**Forward**	**Reverse**
Acc1	GCCTCTTCCTGACAAACGAG	TGACTGCCGAAACATCTCTG
Atgl	TCCTTAGGAGGAATGGCCTAC	TCCTCTTCCTGGGGGACAAC
Fasn	TGGTGAATTGTCTCCGAAAAG	CACGTTCATCACGAGGTCATG
G6p	ACACCGACTACTACAGCAACAG	CCTCGAAAGATAGCAAGAGTAG
Nppa	CTGCTTCGGGGGTAGGATTG	TTCGGTACCGGAAGCTGT
Nppb	GCTGCTTTGGGCACAAGATA	ACAACAACTTCAGTGCGTTACAG
Ppara	AGTTCACGCATGTGAAGGCTG	TGTTCCGGTTCTTCTTCTGAATC
Il6	TCCAGTTGCCTTCTTGGGAC	GTGTAATTAAGCGCCGACTTG
Il10	GCTCTTACTGACTGGCATGAG	CGCAGCTCTAGGAGCATGTG
Mcp1	ACTGAAGCCAGCTCTCTCTTCCTC	TTCCTTCTTGGGGTCAGCACAGAC
Il1β	CAGGCAGGCAGTATCACTCA	AGGCCACAGGTATTTTGTCG
Atf4	GAGCTTCCTGAACAGCGAAGTG	TGGCCACCTCCAGATAGTCATC
Xbp1-s	TGAGAACCAGGAGTTAAGAACACGC	CCTGCACCTGCTGCGGAC
Xbp1-us	TGAGAACCAGGAGTTAAGAACACGC	CCTGCACCTGCTGCGGAC

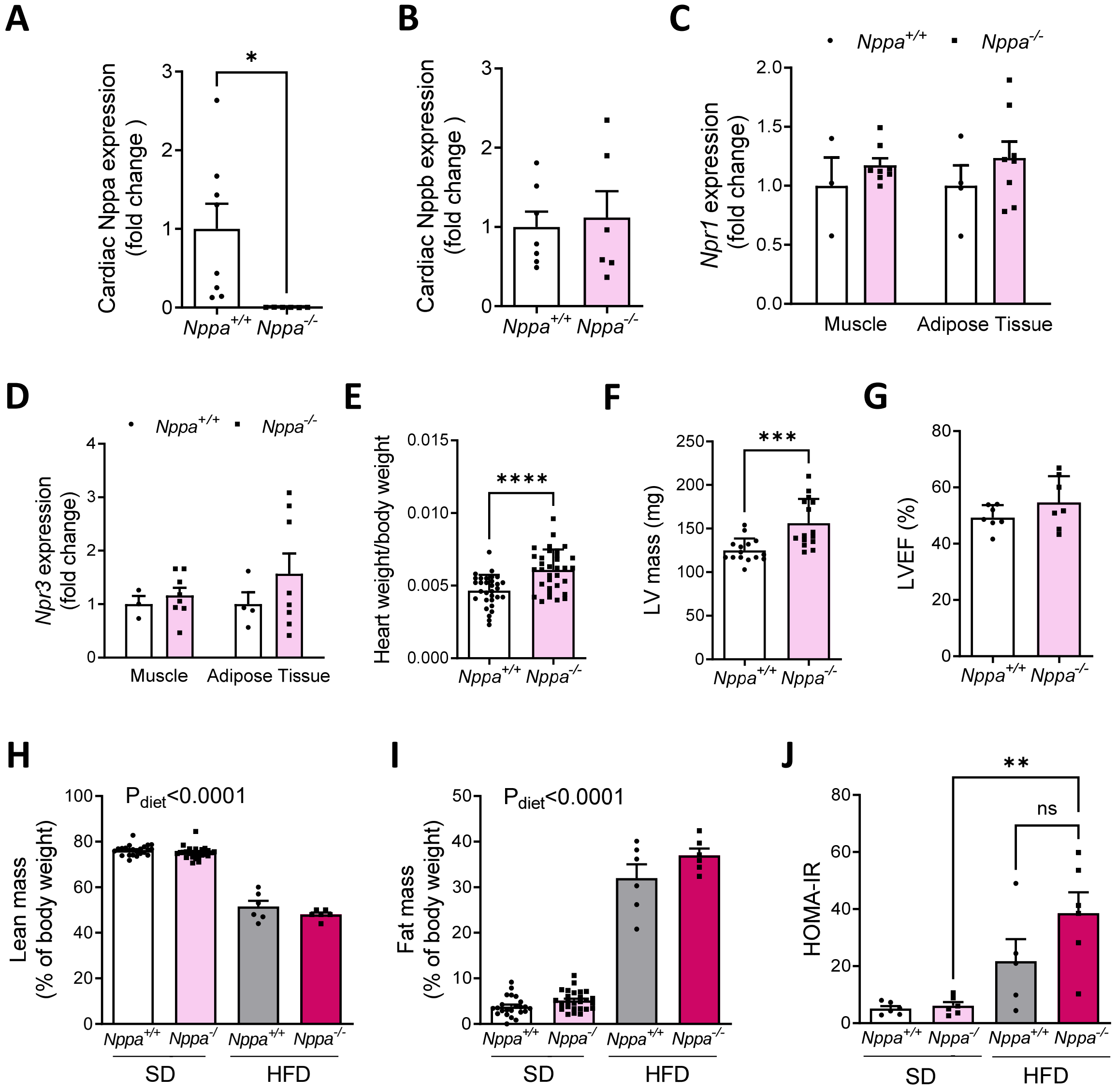

Fig. S1. ANP deficiency promotes insulin resistance in mice

Cardiac ANP (*Nppa*) (**A**) and BNP (*Nppb*) (**B**) gene expression in *Nppa*$^{-/-}$ and *Nppa*$^{+/+}$ mice (n=6-8). *Npr1* (**C**) and *Npr3* (**D**) gene expression in skeletal muscle and adipose tissue of *Nppa*$^{-/-}$ and *Nppa*$^{+/+}$ mice (n=4-8). Heart weight to body weight ratio (n=32-33) (**E**), left ventricular (LV) mass (**F**) and left ventricular ejection fraction (n=7) (**G**) in *Nppa*$^{-/-}$ and *Nppa*$^{+/+}$ mice. Percentage of lean mass (**H**) and fat mass (**I**), and HOMA-IR (**J**) of *Nppa*$^{-/-}$ and *Nppa*$^{+/+}$ mice fed a SD (n=22-26) or HFD (n=6-7). * p≤0.05, **** p<0.0001, versus *Nppa*$^{+/+}$ mice.

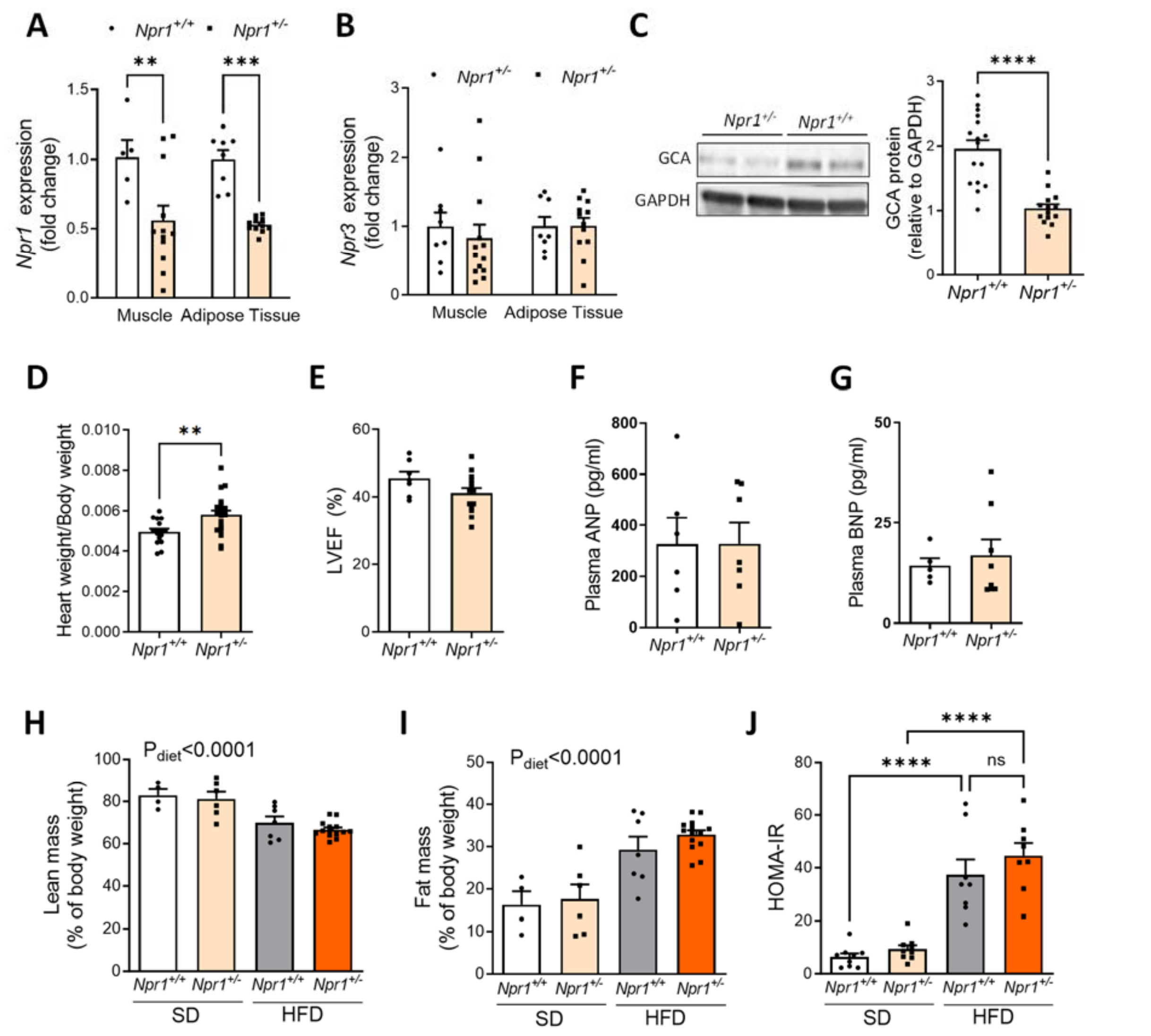

Fig. S2. GCA haploinsufficiency exacerbates HFD-induced insulin resistance

Npr1 (**A**) and *Npr3* (**B**) gene expression in skeletal muscle and adipose tissue of *Npr1*$^{+/-}$ and *Npr1*$^{+/+}$ mice (n=5-12). Representative blot and quantification of GCA protein content (**C**) in EDL muscle of of *Npr1*$^{+/-}$ and *Npr1*$^{+/+}$ mice (n=14-16). Heart weight to body weight ratio (n=16-22) (**D**), left ventricular ejection fraction (n=7-14) (**E**), plasma ANP (**F**) and BNP (**G**) levels (n=5-8) in *Npr1*$^{+/-}$ and *Npr1*$^{+/+}$ mice. Percentage of lean mass (**H**), fat mass (**I**), and HOMA-IR (**J**) of *Npr1*$^{+/-}$ and *Npr1*$^{+/+}$ mice fed a SD (n=4-6) or HFD (n=8-14). ** p<0.01, *** p<0.001, **** p<0.0001, versus *Npr1*$^{+/+}$ mice.

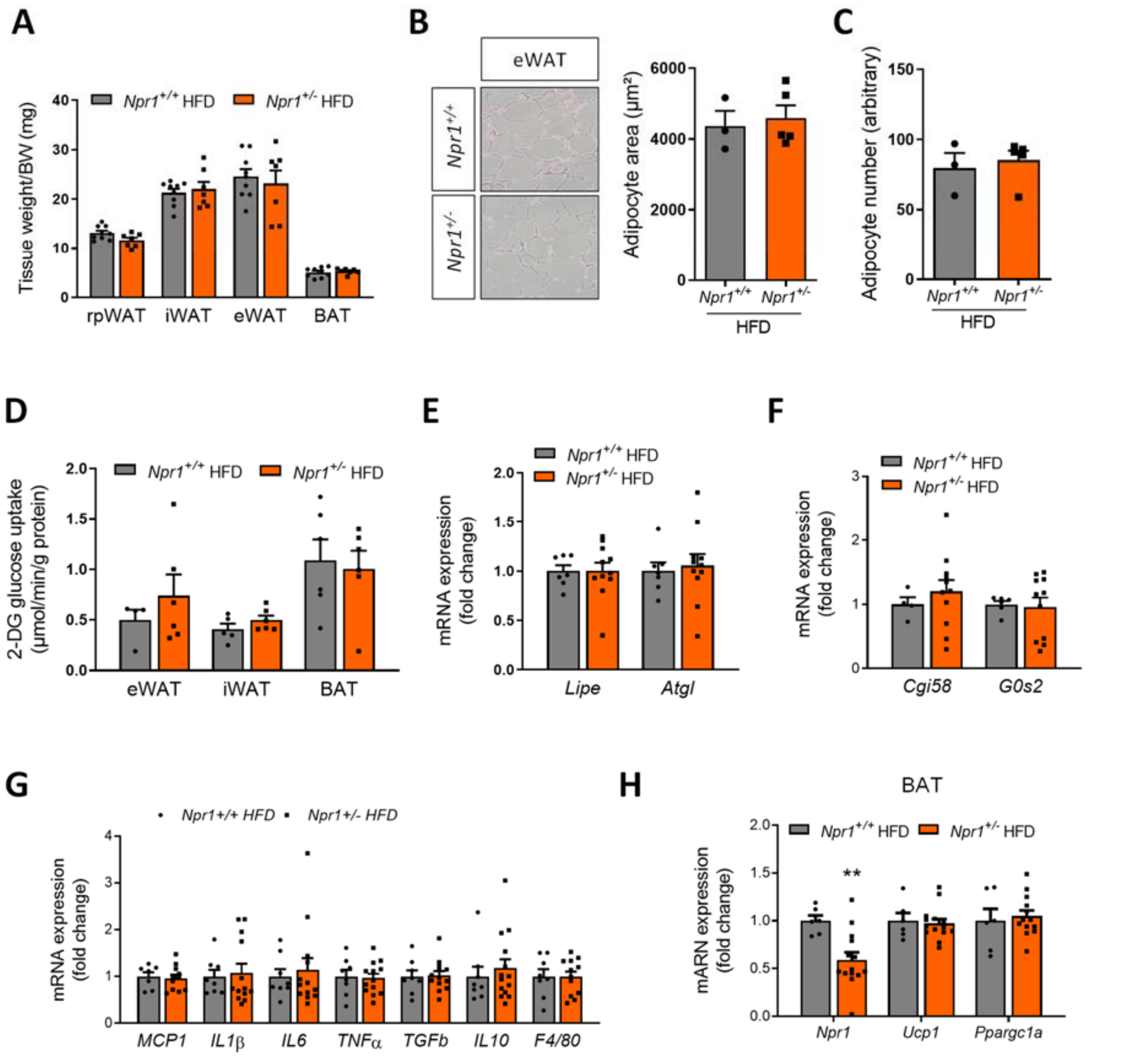

Fig. S3. Impaired glucose homeostasis in GCA haploinsufficient mice is associated with marginal changes in adipose tissue

Ratio of adipose tissues (epidydimal, inguinal, retroperitoneal WAT and BAT) weight to body weight in *Npr1*$^{+/-}$ and *Npr1*$^{+/+}$ HFD-fed mice (n=7-9) (**A**). Representative histological images and adipocytes area (**B**) and number (**C**) in the eWAT of *Npr1*$^{+/-}$ and *Npr1*$^{+/+}$ HFD-fed mice (n=3-5). 2DG-glucose uptake induced by insulin in eWAT, iWAT and BAT of *Npr1*$^{+/-}$ and *Npr1*$^{+/+}$ HFD-fed mice (n=6) (**D**). *Lipe*, *Atgl* (**E**) *and Cgi58, G0s2* (**F**) mRNA expression in WAT of *Npr1*$^{+/-}$ and *Npr1*$^{+/+}$ HFD-fed mice (n=7-11). (**G**) Inflammation markers mRNA expression in eWAT of *Npr1*$^{+/-}$ and *Npr1*$^{+/+}$ HFD-fed mice (n=7-14). (**H**) *Npr1* and thermogenic

gene expression in BAT of *Npr1*$^{+/-}$ and *Npr1*$^{+/+}$ HFD-fed mice (n=6-14). ** $p<0.01$, versus *Npr1*$^{+/+}$ mice.

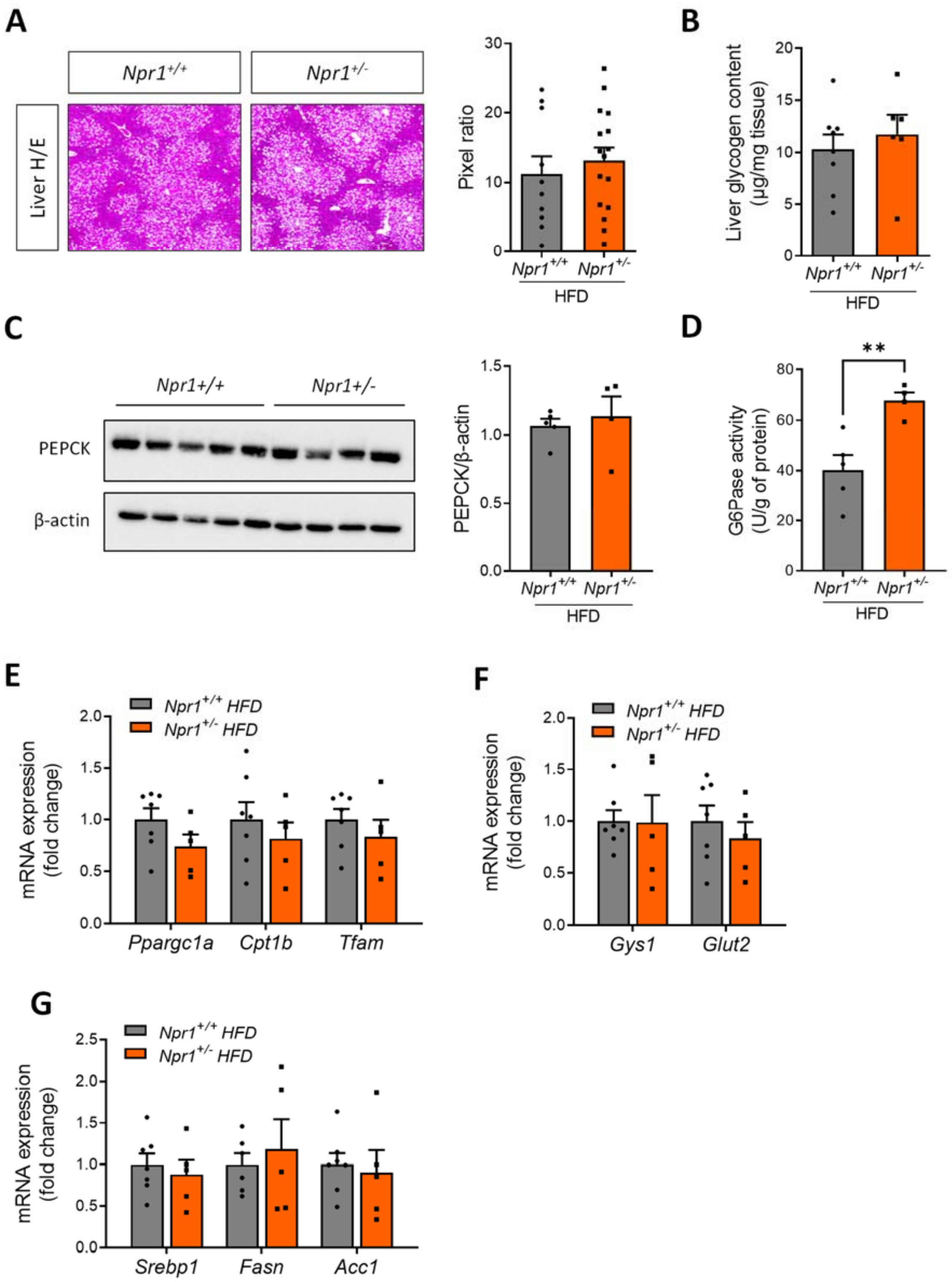

Fig. S4. Impaired glucose homeostasis in GCA haploinsufficient mice is associated with marginal changes in liver

Representative images of liver H&E staining of *Npr1*$^{+/-}$ and *Npr1*$^{+/+}$ HFD-fed mice and quantification of lipid content (n=10-16) (**A**). Liver glycogen content (n=6-8) (**B**),

Representative immunoblots and quantification of PEPCK protein content in liver (**C**) and liver G6Pase activity (n=4-5) (**D**) of *Npr1*$^{+/-}$ and *Npr1*$^{+/+}$ HFD-fed mice. Relative gene expression of markers of lipid oxidation (**E**), glucose metabolism (**F**) and *de novo* lipogenesis (**G**) in liver of *Npr1*$^{+/-}$ and *Npr1*$^{+/+}$ HFD-fed mice (n=5-7). ** $p<0.01$, versus *Npr1*$^{+/+}$ mice.

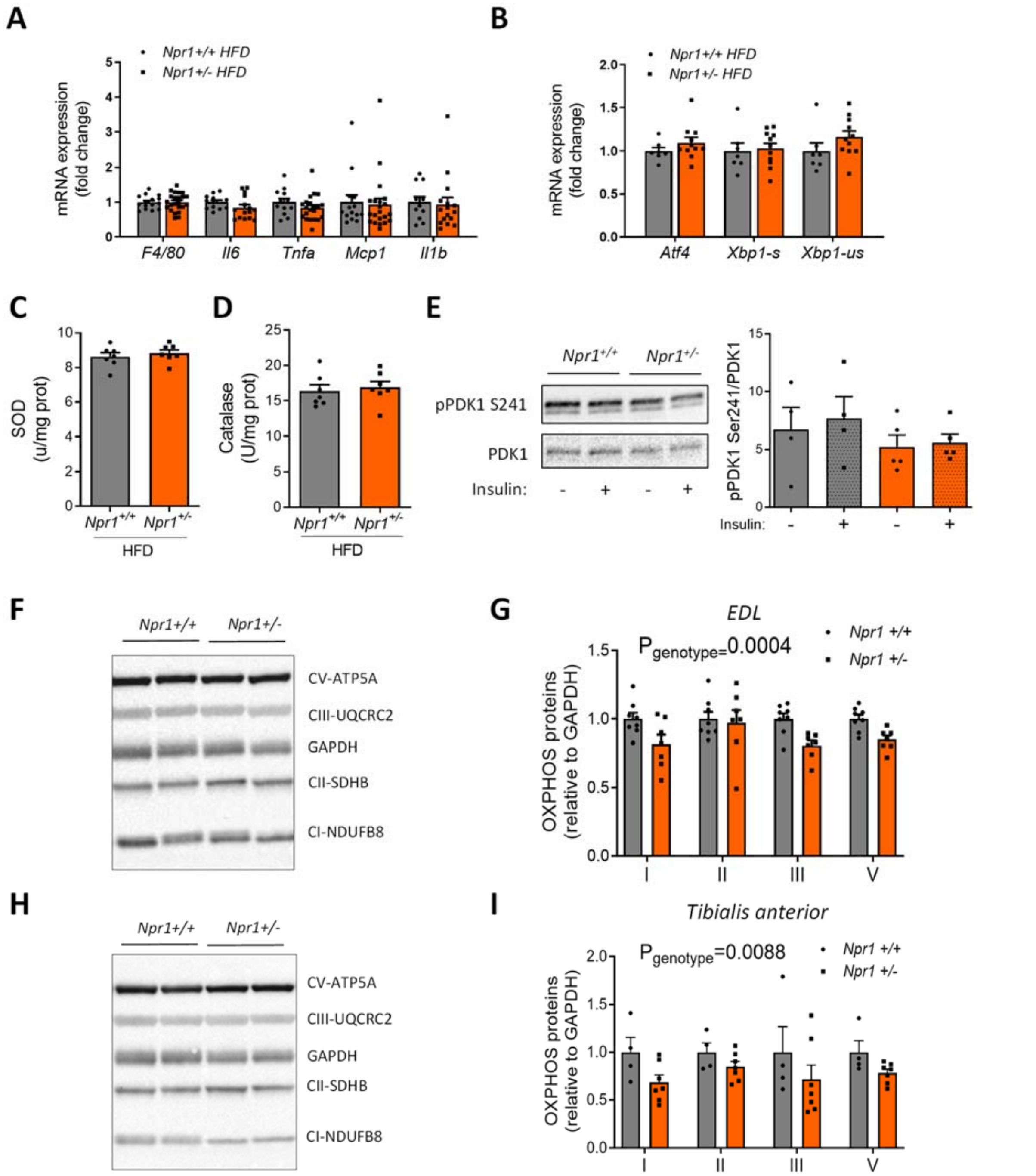

Fig. S5. GCA haploinsufficiency induces mitochondrial dysfunction in skeletal muscle

Relative gene expression of *F4/80, Il6, Tnfa, Mcp1* and *Il1β* in skeletal muscle of *Npr1*$^{+/-}$ and *Npr1*$^{+/+}$ HFD-fed mice (n=14-23) (**A**). *Atf4, Xbp1-s* and *Xbp1-us* mRNA expression in the skeletal muscle of *Npr1*$^{+/-}$ and *Npr1*$^{+/+}$ HFD-fed mice (n=14-23) (**B**). Superoxide dismutase (SOD) (**C**) and catalase (CAT) (**D**) enzyme activity in skeletal muscle of *Npr1*$^{+/-}$ and *Npr1*$^{+/+}$ HFD-fed mice. (**E**) Representative blot and relative quantification of PDK1 phosphorylation at

Ser241 in EDL in absence (-) or presence (+) of insulin. Representative blot and relative quantification of OXPHOS protein complex in *extensor digitorum longus* (EDL) (**F-G**) and *tibialis anterior* (**H-I**) muscle of $Npr1^{+/-}$ and $Npr1^{+/+}$ HFD-fed mice. * p≤0.05, versus $Npr1^{+/+}$ mice.

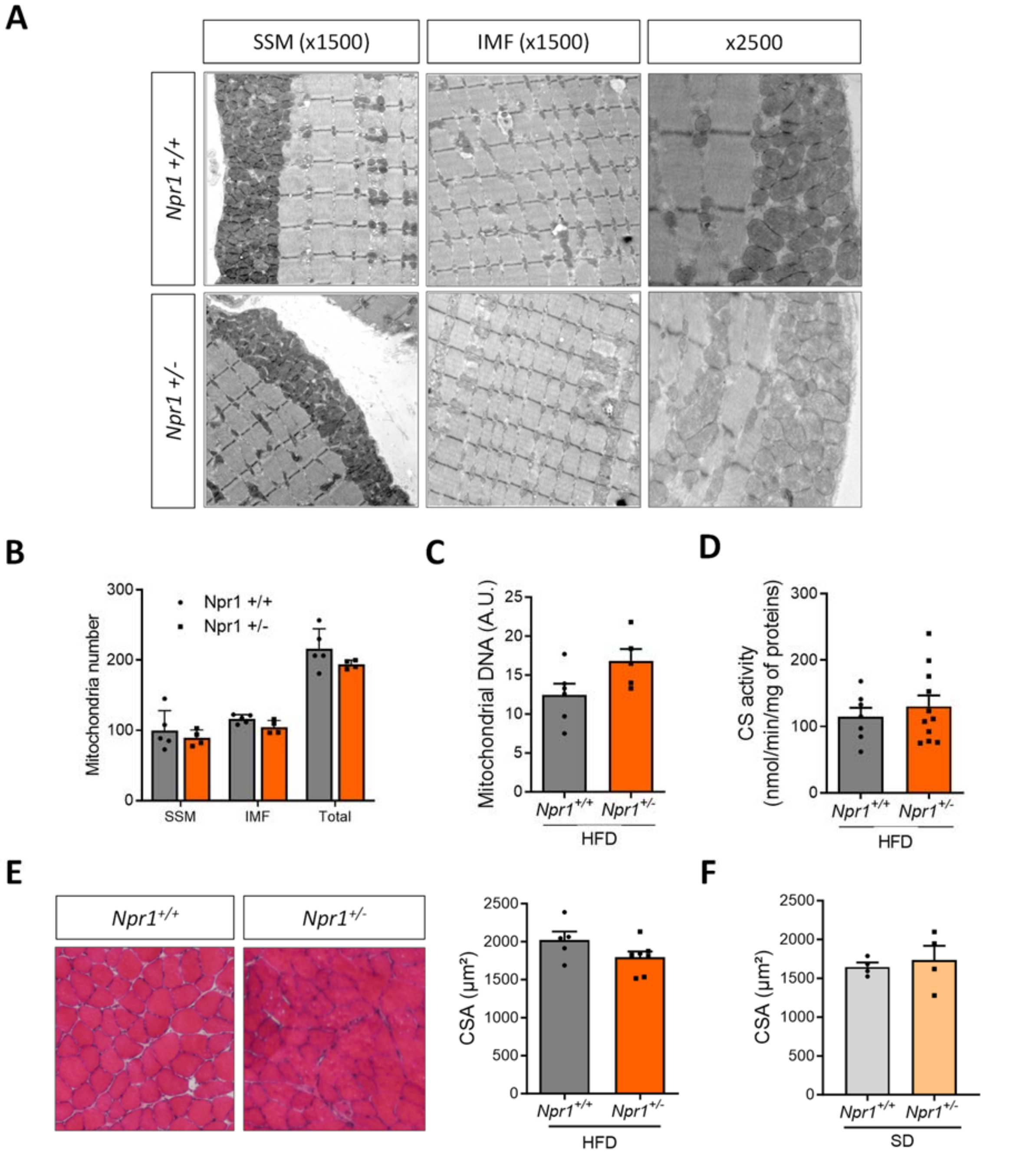

Fig. S6. GCA haploinsufficiency does not alter mitochondrial mass in skeletal muscle. Representative electron micrograph images of subsarcolemal (SSM) and intermyofibrillar (IMF) mitochondria at x1500 and x2500 magnification (**A**), number quantification (**B**) (n=5), mitochondrial DNA content (n=5-6) (**C**), citrate synthase activity in whole skeletal muscle (n=7-11) (**D**), and representative H/E staining and quantification of mean fiber CSA (**E**) of *Npr1*$^{+/-}$ and *Npr1*$^{+/+}$ mice fed a HFD. Mean fiber CSA in *Npr1*$^{+/-}$ and *Npr1*$^{+/+}$ mice fed a SD (**F**).

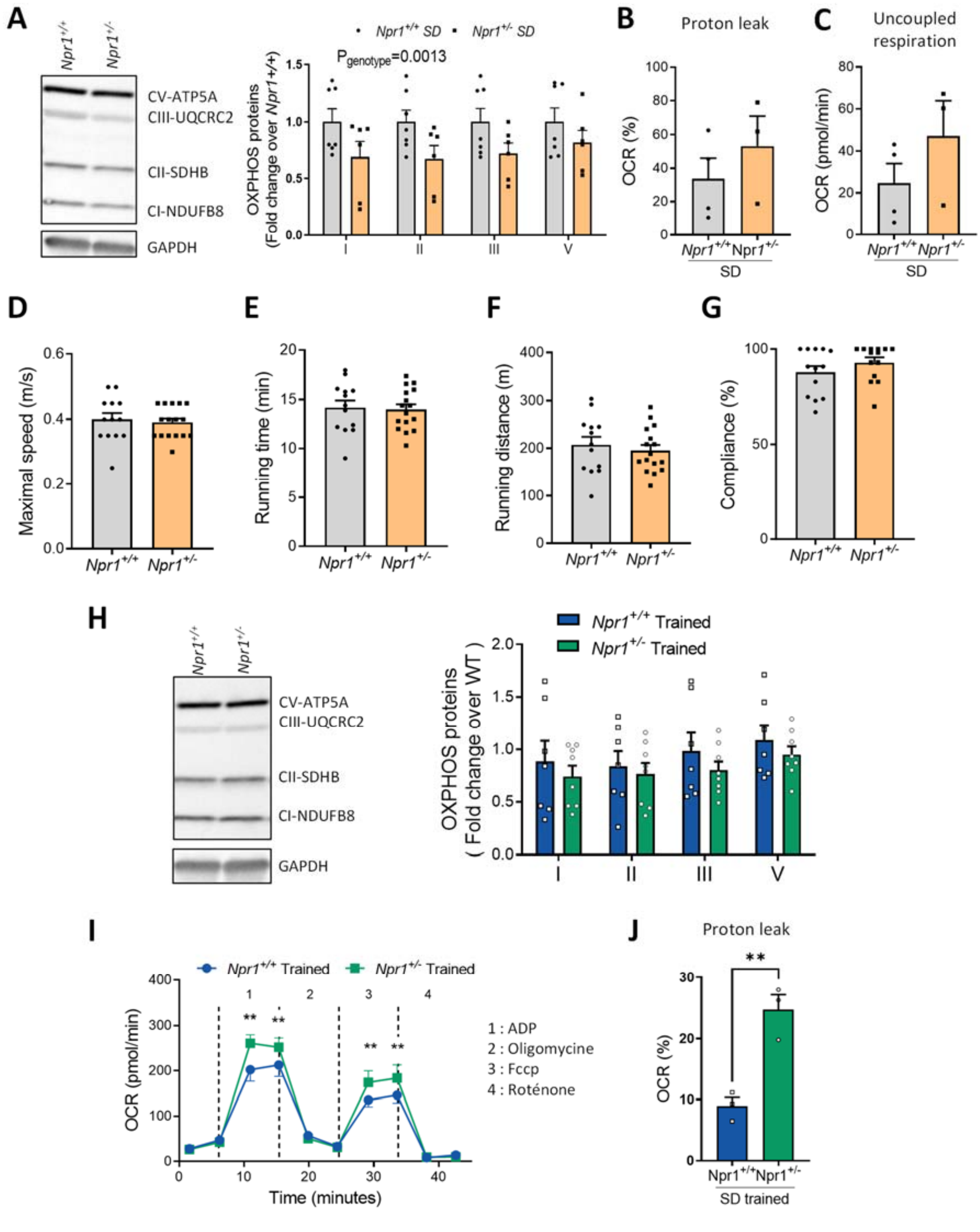

Fig. S7. Impaired endurance capacity and adaptations to training in GCA haploinsufficient mice

Representative blot and relative quantification of OXPHOS complex protein content in EDL muscle (n=6-7) (**A**), mitochondrial proton leak (**B**) and uncoupled respiration (n=3) (**C**) of isolated mitochondria from *gastrocnemius* muscle of *Npr1*$^{+/-}$ and *Npr1*$^{+/+}$ sedentary mice under

SD. Maximal speed (**D**), running time (**E**), running distance (**F**) during a maximal speed incremental test in *Npr1*$^{+/-}$ and *Npr1*$^{+/+}$ sedentary mice under SD (n=13-16). Compliance of *Npr1*$^{+/-}$ and *Npr1*$^{+/+}$ sedentary mice under SD during 4-weeks of endurance training (**G**). Representative blot and relative quantification of OXPHOS protein content in EDL muscle (n=6-7) (**H**), mitochondrial oxygen consumption rate (OCR) (**I**) and proton leak (n=3) (**J**) of isolated mitochondria from *gastrocnemius* muscle of *Npr1*$^{+/-}$ and *Npr1*$^{+/+}$ 4-week trained mice under SD. ** $p<0.01$, versus *Npr1*$^{+/+}$ mice.

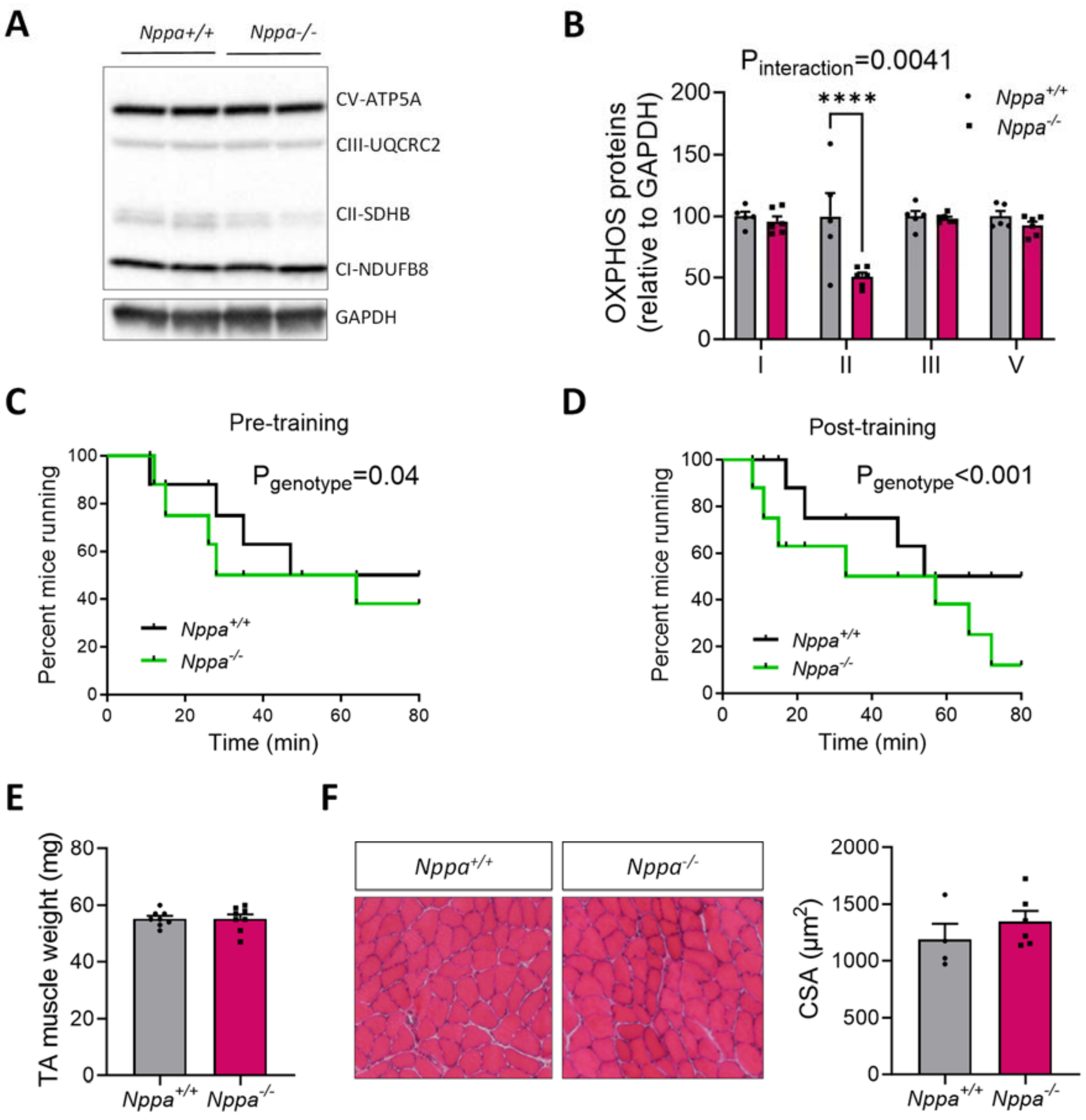

Fig. S8. Impaired endurance capacity and adaptations to training in ANP-deficient mice

Representative blot (**A**) and relative quantification (**B**) of OXPHOS protein complex in *soleus* muscle of HFD-fed *Nppa*$^{+/+}$ and *Nppa*$^{-/-}$ mice. Endurance running capacity prior to (**C**) and following (**D**) a 4-week of endurance training in *Nppa*$^{+/+}$ and *Nppa*$^{-/-}$ under SD. *Tibialis Anterior* muscle weight (**E**), and representative H/E staining and quantification of mean fiber CSA (**F**) in *Npr1*$^{+/-}$ and *Npr1*$^{+/+}$ mice fed a HFD.

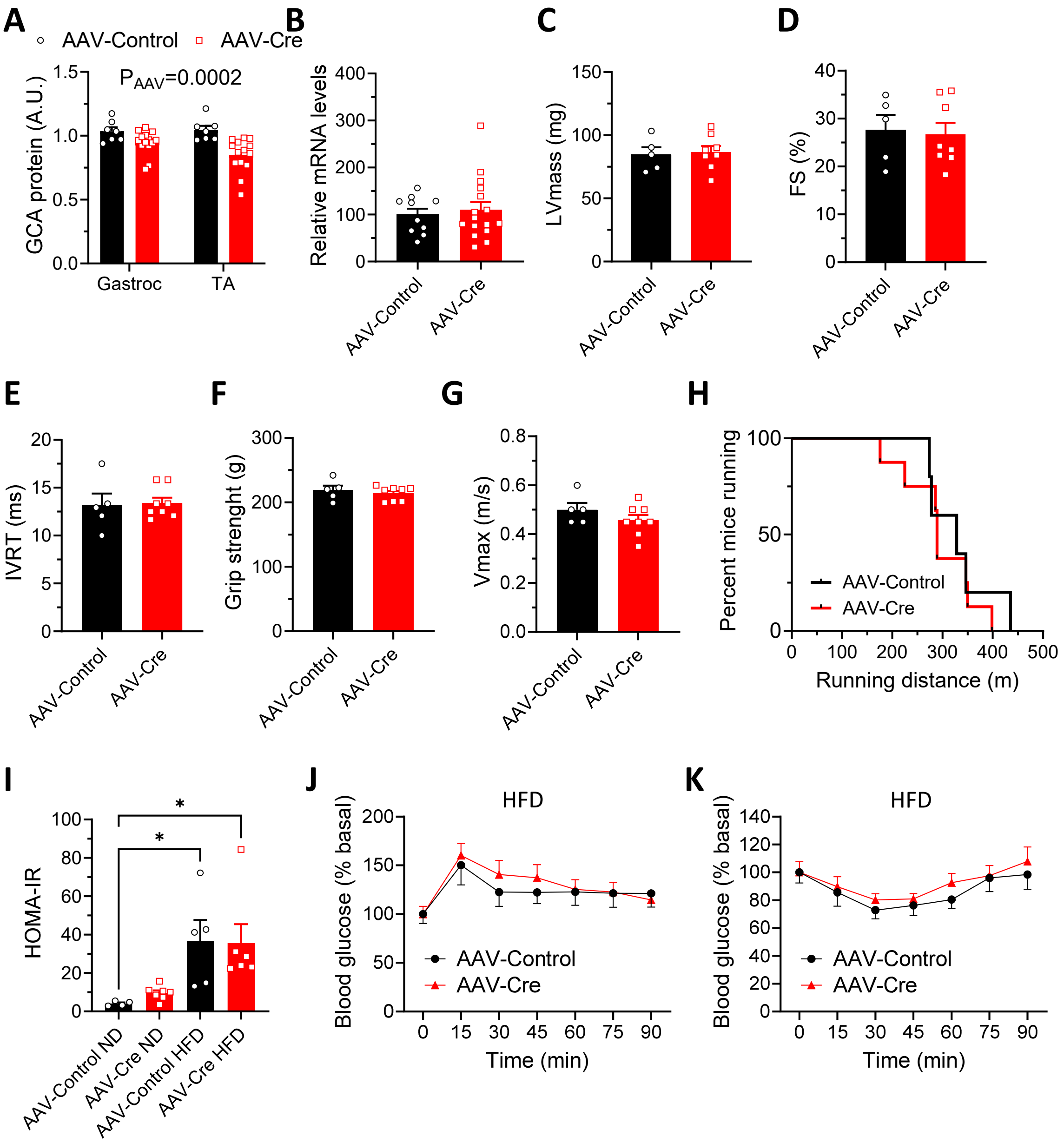

Fig. S9. Mice with partial loss of GCA signaling specifically in skeletal muscle

Representative blot and relative quantification of GCA protein in *gastrocnemius* and *tibialis anterior* muscle (**A**) of *Gca* fl/fl male mice injected with AAV-Control or AAV-Cre under standard chow diet. Relative Gca mRNA levels (**B**), left ventricular (LV) mass (**C**), fractional shortening (FS) (**D**) and isovolumetric relaxation time (IVRT) (**E**) in *Gca* fl/fl male mice injected with AAV-Control (n=5) or AAV-Cre (n=8). Grip strength (**F**), maximal speed (Vmax) (**G**) and percent running mice during the Vmax (**H**) in *Gca* fl/fl male mice injected with AAV-

Control (n=5) or AAV-Cre (n=8). HOMA-IR (**I**), blood glucose levels during a GTT (**J**) and an ITT (**K**) in *Gca* fl/fl male HFD-fed mice injected with AAV-Control or AAV-Cre.

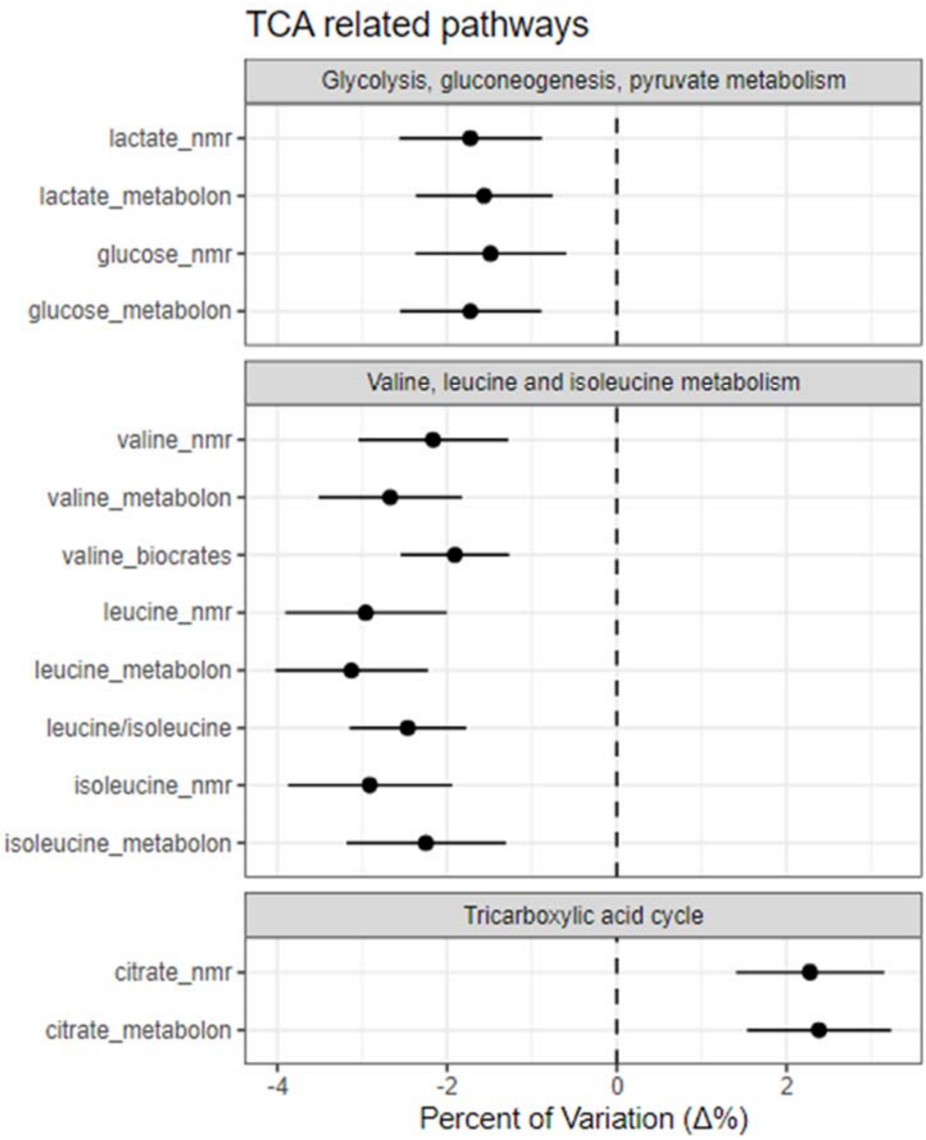

Fig. S10. Associations between serum MR-proANP and metabolites in the KORA cohort

Forest plot of significant correlations between serum MR-proANP and various metabolites of the TCA cycle from the KORA cohort of the NMR, Biocrates, and Metabolon dataset. Dots depict the mean % variation and lines the 95% confidence interval.
